# Temporal evolution of cellular heterogeneity during the progression to advanced AR-negative prostate cancer

**DOI:** 10.1038/s41467-021-23780-y

**Published:** 2021-06-07

**Authors:** Nicholas J. Brady, Alyssa M. Bagadion, Richa Singh, Vincenza Conteduca, Lucie Van Emmenis, Elisa Arceci, Hubert Pakula, Ryan Carelli, Francesca Khani, Martin Bakht, Michael Sigouros, Rohan Bareja, Andrea Sboner, Olivier Elemento, Scott Tagawa, David M. Nanus, Massimo Loda, Himisha Beltran, Brian Robinson, David S. Rickman

**Affiliations:** 1 Department of Pathology and Laboratory Medicine, Weill Cornell Medicine, New York, NY USA; 2 Department of Medical Oncology, Dana Farber Cancer Institute, Boston, MA USA; 3 Department of Urology, Weill Cornell Medicine, New York, NY USA; 4 Caryl and Israel Englander Institute for Precision Medicine, New York-Presbyterian Hospital, Weill Cornell Medicine, New York, NY USA; 5 Meyer Cancer Center, Weill Cornell Medicine, New York, NY USA; 6 Department of Physiology and Biophysics, Weill Cornell Medicine, New York, NY USA; 7 Institute for Computational Biomedicine, Weill Cornell Medicine, New York, NY USA; 8 Department of Medicine, Weill Cornell Medicine, New York, NY USA

**Keywords:** Prostate cancer, Epigenetics, Gene expression, Transcriptomics

## Abstract

Despite advances in the development of highly effective androgen receptor (AR)-directed therapies for the treatment of men with advanced prostate cancer, acquired resistance to such therapies frequently ensues. A significant subset of patients with resistant disease develop AR-negative tumors that lose their luminal identity and display neuroendocrine features (neuroendocrine prostate cancer (NEPC)). The cellular heterogeneity and the molecular evolution during the progression from AR-positive adenocarcinoma to AR-negative NEPC has yet to be characterized. Utilizing a new genetically engineered mouse model, we have characterized the synergy between *Rb1* loss and *MYCN* (encodes N-Myc) overexpression which results in the formation of AR-negative, poorly differentiated tumors with high metastatic potential. Single-cell-based approaches revealed striking temporal changes to the transcriptome and chromatin accessibility which have identified the emergence of distinct cell populations, marked by differential expression of *Ascl1* and *Pou2f3*, during the transition to NEPC. Moreover, global DNA methylation and the N-Myc cistrome are redirected following *Rb1* loss. Altogether, our data provide insight into the progression of prostate adenocarcinoma to NEPC.

## Abstract (web summary)

The heterogeneity of tumor evolution from AR-positive, adenocarcinoma to AR-negative, neuroendocrine prostate cancer (NEPC) is not fully characterized. Here the authors generate a mouse model to show that Rb1 loss and MYCN overexpression accelerates the progression to AR-negative NEPC and identify emergence of distinct subpopulations of NEPC cells.

## Introduction

Despite advances in the development of highly effective androgen receptor (AR)-targeted therapies for the treatment of men with advanced prostate cancer acquired resistance ultimately ensues. A significant subset of patients with the treatment-resistant disease develops AR-negative prostate tumors that lose their luminal identity and display neuroendocrine features (neuroendocrine prostate cancer (NEPC)^1–3^). NEPC is characterized by loss of AR and luminal (cytokeratins 8 and 18 (CK8, CK18)) epithelial markers, as well as expression of basal (CK14, CK5) epithelial markers, pluripotency (Sry-box 2, SOX2), neuroendocrine markers (Synaptophysin (SYP), Chromogranin A (CHGA), Neuron Specific Enolase (NSE)), and genes associated with the neural lineage^4^. Although NEPC tumors arise clonally from castration-resistant prostate adenocarcinoma (CRPC) and share genomic alterations, there is significant epigenetic deregulation during the progression from CRPC towards NEPC^2^. We and others previously identified N-Myc (encoded by *MYCN*) as a key driver of this progression^5–8^. Changes in the N-Myc cistrome, its interacting co-factors, and N-Myc-directed epigenomic reprogramming are androgen-dependent and drive a lineage switch in prostate cancer epithelial cells towards a neural identity that favors the development of AR independence and NEPC^5–7^. Loss of *TP53* and *RB1*^9–15^, and upregulation of enhancer of zeste homolog 2 (EZH2^7,10^) have also been associated with the development of AR-negative prostate cancer. However, little is known about how aberrant N-Myc expression cooperates with specific genomic events such as loss of *TP53*, and *RB1* and other molecular alterations to promote the acquisition of the neuroendocrine phenotype and the heterogeneity of tumor evolution towards NEPC from CRPC in the absence of androgen.

In both CRPC and NEPC, *MYCN* overexpression and *RB1* loss can occur independently or co-occur in patient samples. Mouse models of retinoblastoma and neuroblastoma show that *Rb1* loss in *MYCN*-driven tumors accelerates tumor progression and shortens survival time^16,17^. The clinical and biological significance of this co-occurrence in prostate cancer has not been described. In this study, we describe a synergy between *MYCN* overexpression and *RB1* loss in driving AR-negative, poorly differentiated metastatic prostate cancer and a molecular program that is associated with clinical NEPC.

## Results

### Tumors with high *MYCN* expression and *RB1* deletion are associated with a worse prognosis

**Fig. 1:**
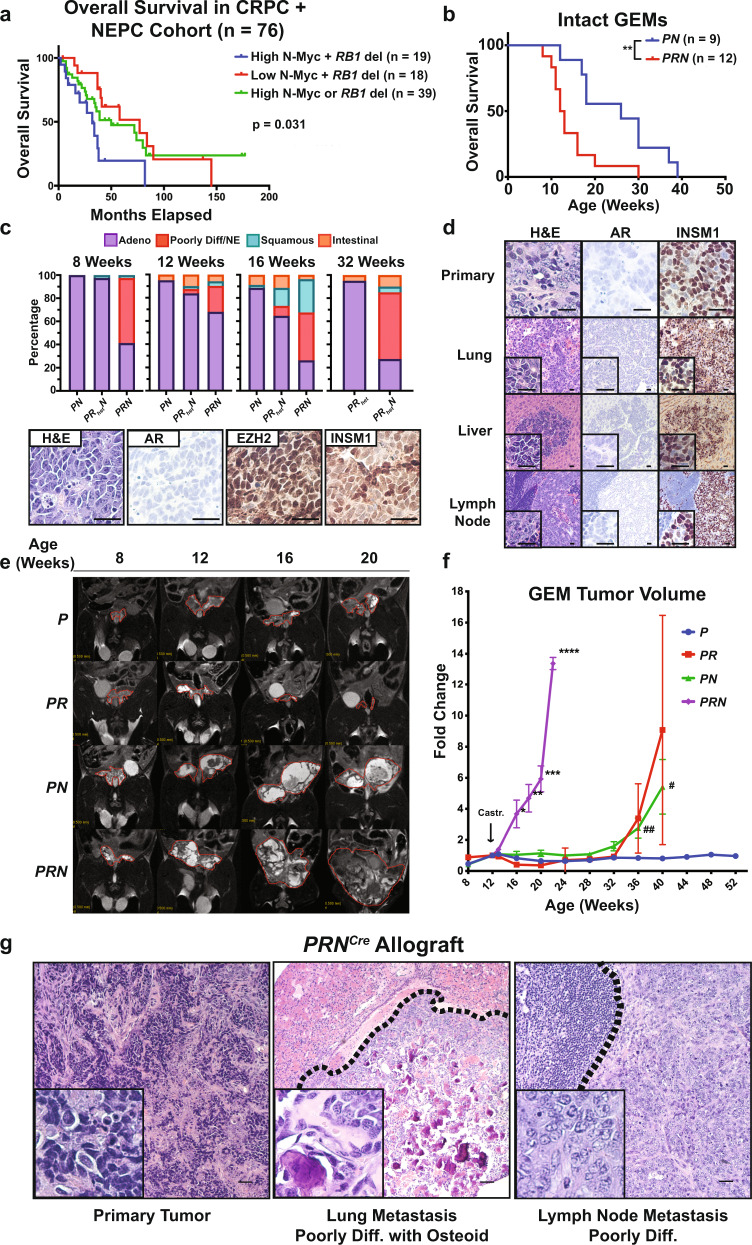
*MYCN*+ and *RB1* deletion accelerate progression to NEPC. **a** Kaplan-Meier plots of CRPC (*n* = 55) and NEPC (*n* = 21) patients stratified into three categories according to *MYCN* (N-Myc) expression and/or *RB1* status. Univariate overall survival analysis was calculated from the time of initial diagnosis of metastatic disease to death from any cause. Patients still alive at the time of the last follow-up were censored. Patients with high N-Myc expression and *RB1* deletion showed significantly worse survival compared to the other two groups (*p* = 0.031, Kaplan-Meier estimator (two-sided log-rank test)). **b** Survival plots of intact *PN* or *PRN* mice using the Kaplan-Meier estimator (**p = 0.0056, log-rank test). **c** Top: percentage of GEM tumor foci with conventional adenocarcinoma (including HGPIN, intracystic carcinoma, adenocarcinoma), poorly differentiated/neuroendocrine features, squamous differentiation, and intestinal differentiation based on pathologist assessment. Bottom: Hematoxylin and eosin (H&E) and IHC staining of NEPC focus for N-Myc, RB1, epithelial markers AR and CK8, reprogramming factor EZH2, and small cell neuroendocrine marker INSM1. Images are representative of 13 mice. Scale bar: 50 μm. **d** H&E and IHC of AR, N-Myc, INSM1 on primary tumor, lung, liver, and lymph node metastasis in a 19-week-old *PRN* mouse. Images are representative of 2 mice. Scale bar: 50 μm. **e** MRI coronal scans from *P* (*n* = 5), *PR* (*n* = 3); *PN* (*n* = 4); and *PRN* (n = 6) GEMs from 8 to 20 weeks of age. Mice were castrated at 12 weeks of age. **f** Fold change volume over time of *P* (*n* = 5), *PR* (*n* = 3); *PN* (*n* = 4); and *PRN* (*n* = 6) GEM tumors from mice castrated at 12 weeks. PRN: *p = 0.0187, **p = 0.0027, ***p = 0.0002, ****p < 0.0001, PN: ^#^*p* = 0.0129, ^##^*p* = 0.0075, two-tailed *t*-test between indicated genotype and *P* mice. Data are presented as mean values +/− SEM. **g** H&Es of poorly differentiated subcutaneous mouse allograft primary tumor and metastases derived from Adeno-Cre-transduced *PRN* organoids. The dashed lines separate the tumor metastasis from normal tissue. Images are representative of 18 independent mice. Scale bar: 50 μm.

To determine if the co-occurrence of *MYCN* overexpression and *RB1* loss is clinically meaningful, we assessed overall survival differences. Seventy-six patient biopsies were grouped according to their tumor histological classification (CRPC or NEPC) and stratified into three categories based on RNA-seq values of *MYCN* expression (high vs. low) and *RB1* genomic status (deletion, yes vs. no). Of the 76 patient samples, 55 were histologically classified as CRPC and 21 as NEPC. We observed that 19/76 (25%) of patients had tumors with high N-Myc expression and *RB1* deletion. Interestingly, 9/55 (16.4%) CRPC and 10/21 (47.6%) NEPC had high N-Myc expression and *RB1* deletion, suggesting that co-occurrence of N-Myc overexpression and *RB1* loss may be enriched for during the development of NEPC (Fig. 1a and Supplementary Fig. 1a). Moreover, we observed that high N-Myc expression in combination with *RB1* deletion in patient CRPC and NEPC tumors was significantly associated with worse overall survival compared to those with high N-Myc expression/*RB1* normal or low N-Myc expression/*RB1* normal (19.93 months (range between 1.83 and 81.5 months) vs. 32.27 (6.6–87.17) or 42.90 (4.2–145.3) months, *p* = 0.03, Fig. 1a and Supplementary Fig. 1a). Our data showed that the co-occurrence of both high N-Myc expression and *RB1* deletion predicted an overall worse prognosis. Several studies have investigated the role of N-Myc overexpression^5–8^ or *RB1* loss^10,12^ in driving the development of NEPC, however, the possible synergistic role, if any, of these genetic alterations as drivers has not been reported.

### *MYCN*+ and *Rb1* deletion accelerates progression to AR-negative, poorly differentiated/NEPC tumors, metastases, and decreased median survival

To determine the impact of N-Myc overexpression and *Rb1* loss on tumor progression in vivo, we introduced *Rb1*^*f/f*^ alleles into our previously described *Pb-Cre4*^*+/−*^*;Pten*^*f/f*^*;LSL-MYCN*^*+/+*^ genetically engineered mouse (GEM) model^7^. For simplicity, we will refer to the different genotypes and controls with aliases (Supplementary Fig. 1b). Similar to the clinical cases, both *Pten*^*f/f*^*;Rb1*^*f/+*^*;MYCN* + (*PR*_*het*_*N*) and *Pten*^*f/f*^*;Rb1*^*f/f*^*;MYCN* + (*PRN*) mice had a poorer survival (median survival time of 12.5 weeks (*PRN* mice) or 19 weeks (*PR*_*het*_*N* mice)) compared to *Pten*^*f/f*^*;MYCN* + (*PN*) mice (median survival time of 26 weeks) and *Pten*^*f/f*^*;Rb1*^*f/f*^ (*PR*) mice (38 weeks, Fig. 1b and Supplementary Fig. 1c). In addition, *PRN* and *PR*_*het*_*N* mice developed large, invasive tumors with AR-negative and poorly differentiated foci as early as 8 weeks (*PRN*) or 12 weeks (*PR*_*het*_*N*). These regions showed little resemblance to conventional adenocarcinoma but were negative for NE markers, potentially as a transition between conventional adenocarcinoma and NEPC states. Some of these foci, however, were positive for the neuroendocrine marker INSM1^18,19^, EZH2, as well as NKX2-1, a marker associated with small cell neuroendocrine carcinomas (including prostate)^18–20^ (Fig. 1c, d and Supplementary Fig. 1d, e). Based on hematoxylin and eosin (H&E) staining, some of these foci displayed condensed nuclei and scant cytoplasm, characteristics of NEPC morphology. These tumors also included other foci of divergent differentiation (i.e., intestinal and squamous), as well as conventional, AR-positive, adenocarcinoma similar to primary tumors from *PN* mice previously described^6,7^ but the percentage of conventional adenocarcinoma decreased over time while the percentage of poorly differentiated histology increased over time. This is in contrast to tumors from *PN* or *PRN* mice, which had primarily high-grade prostatic intraepithelial neoplasia (HGPIN) at the earlier time points.

We also found that the different genotypes were associated with different metastatic potentials (summarized in Supplementary Table 1, Fig. 1d, and Supplementary Fig. 1e–g). Lung and liver metastatic lesions were AR negative, however, lymph node metastatic lesions contained both AR-positive and negative tumor cells, suggesting that loss of AR expression is not necessary for metastasis. In addition, both lung and lymph node lesions were RB1-positive, further suggesting that complete loss of *Rb1* is not necessary to provide metastatic potential. For *PRN* mice, the onset of metastasis occurred as early as 8 weeks and was significantly accelerated compared to *PN*^6,7^ or *PR*^10^ mice. By 12 weeks, 100% of intact *PRN* mice developed distant metastatic lesions (*n* = 8). Similar to primary tumors (Supplementary Fig. 1e, g), metastases to the lung, liver, lymph nodes, and kidney primarily consisted of large and small cell neuroendocrine histologies that stained positive for INSM1 and N-Myc but negative for AR. These data suggest that *Rb1* loss in the context of *PN* mice accelerates the formation of poorly differentiated tumors with high metastatic potential, and eventually NEPC.

To determine if *Rb1* loss promotes castration resistance in the context of *PN* mice, we performed longitudinal magnetic resonance imaging (MRI) analyses to track tumor growth before and after castration at 12 weeks of age. Based on both coronal and axial MRI-based tumor volume measurements, *PRN* mice did not respond to surgical castration and continued to grow rapidly (Fig. 1e, f). In contrast, tumors in *PR*, *PN*, and *Pten*^*f/f*^ (*P*) mice regressed post-castration. As expected, we found that *PN* and *PR* tumors resumed growth 24 weeks post-castration whereas tumors with *Pten* loss alone did not show tumor regrowth at 52 weeks of age, consistent with the previous reports^7,10^. In castrated mice, the median survival time was reduced for *PRN* (24 weeks) compared to *PR* (43.5 weeks) and *PN* (44 weeks) mice (Supplementary Fig. 2a). In addition, post-castration tumors from *PRN* mice harbored foci enriched with divergent differentiated histology compared to adenocarcinoma and increased metastatic potential compared to *PR* and *PN* mice (Supplementary Fig. 2a, b, d).

To determine if the local prostate microenvironment is essential for the observed phenotypes, we derived three-dimensional organoids from normal prostate epithelial tissue obtained from 7 to 8-week old *Pb-Cre4-*negative GEMs and transduced them in vitro with a Cre-expressing adenovirus. Immunoblot and IHC analyses confirmed the presence of functional alleles following Cre induction and confirmed that all GEM-derived organoids were positive for AR (Supplementary Fig. 2c, e). We injected *PN*^*Cre*^ and *PRN*^*Cre*^ organoids subcutaneously into nude mice and monitored allograft tumor growth over time. Tumor volume continuously increased for *PRN*^*Cre*^ allografts (*n* = 20) from week 3 to 6 while *PN*^*Cre*^ allografts (*n* = 5) grew at a significantly slower rate which also translated to a significant difference in tumor weights between the groups (*p* = 0.0003, Supplementary Fig. 2f, g). Moreover, *PRN*^*Cre*^ allograft tumors manifested a similar breakdown and proportion of the different histological patterns to what was observed in primary tumors of *PRN* mice (e.g., AR-positive adenocarcinoma and AR-negative, poorly differentiated foci, Supplementary Fig. 2h, i). Interestingly, allografts from either genotype contained tumor cells that were either AR-negative, weak, or strong, indicating that organoids that were initially AR-positive in vitro gradually lost AR expression in vivo. However, we observed poorly differentiated foci with NEPC morphology primarily in *PRN*^*Cre*^ allografts (Supplementary Fig. 2h, i). *PRN*^*Cre*^ allograft growth rate was slightly reduced following castration (Supplementary Fig. 2j) which is likely due to the fact that the tumors were heterogeneous with a component of AR-responsive adenocarcinoma.

Lastly, lung and lymph node metastatic lesions were only observed in mice bearing *PRN*^*Cre*^ allografts (Fig. 1g). The majority of metastatic lesions were poorly differentiated although some had squamous and osteoid differentiation. Altogether, these data suggest that the *MYCN* overexpression and *Pten/Rb1* loss-induced NEPC morphology, loss of AR expression, and increased metastatic potential occur in a cell-autonomous manner.

### N-Myc and *Rb1* genomic loss drives a molecular program consistent with NEPC

**Fig. 2:**
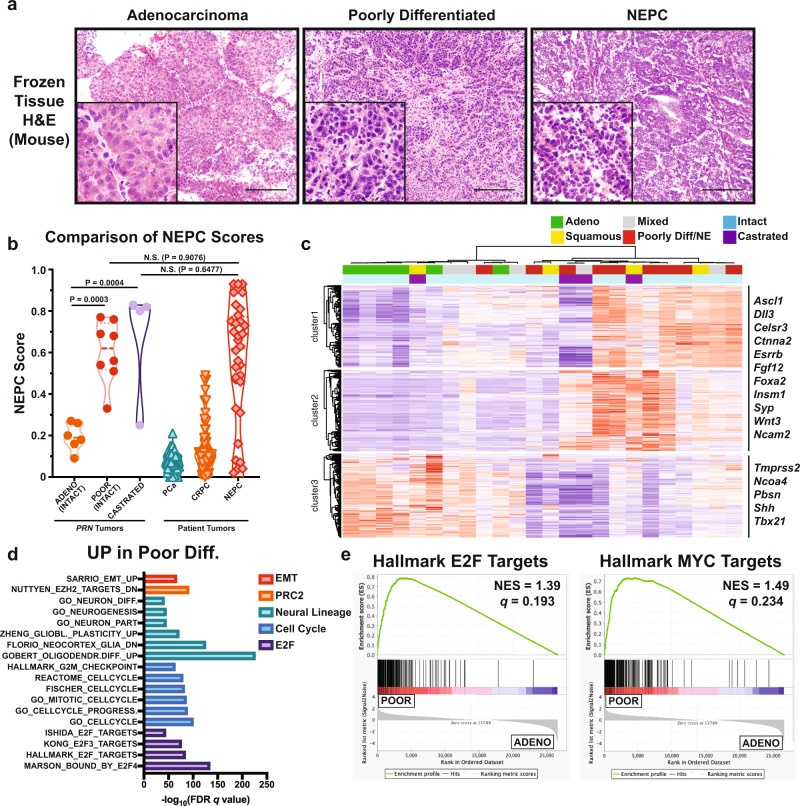
Poorly differentiated tumors have molecular signatures similar to clinical NEPC. **a** H&E from frozen GEM tumors showing examples of adenocarcinoma, poorly differentiated, and NEPC histology. Images are representative of 20 independent mice. Scale bar: 50 μm. **b** Violin plot of NEPC scores from *PRN* GEMs with conventional adenocarcinoma and poorly differentiated/NEPC histologies, as well as PCa, CRPC, and NEPC patient samples. Significance was calculated using one-way ANOVA with Tukey’s multiple comparison test. **c** Heatmap and hierarchical clustering of poor, squamous, adenocarcinoma, and mixed differentiation foci from GEMs based on the *PRN* POOR vs. *PN* ADENO Signature. All castrated mice are of *PRN* genotype. **d** Top gene sets related to E2F targets, cell cycle, neuronal development, epigenetic reprogramming, epithelial identity, p53 targets, and AR signaling. UP: log_2_(FC) >1.5; DOWN: log_2_(FC) <−1.5, Benjamini-Hochberg adjusted two-sided *t*-test *p* < 0.05. **e** GSEA enrichment plots of Hallmark E2F Targets and Hallmark MYC Targets in poorly differentiated (POOR) vs. adenocarcinoma (ADENO).

To determine if *MYCN* expression synergizes with *Rb1* loss to drive a molecular program associated with NEPC, we performed bulk RNA-seq on histologically distinct tumor foci ranging from adenocarcinoma to NEPC (Fig. 2a). Previous studies showed that *TP53* mutations with *RB1* loss drive a phenotype similar to what we observed in the *PRN* mice^10,12,14^. Therefore, we first queried our RNA-seq data for spontaneous mutations in *Trp53* and in *Rb1*. All single nucleotide variants (SNVs) and insertions/deletions (indels) in the *Trp53* and *Rb1* genes were profiled for each histopathological subtype of mouse tumors across all genotypes (Supplementary Fig. 3a). We observed exonic non-silent mutations in *Trp53* exons and synonymous exonic mutations in *Rb1*. Of the *Trp53* alterations, we noted a missense mutation (c.T715G) in exon 7 within the DNA binding domain. This mutation is equivalent to C242R in the human *TP53* gene (obtained from Vista-Point tool^21,22^) and is predicted to have a moderate effect (Ensembl Variant Effect Predictor) to deleterious (SIFT) or damaging (PolyPhen)^23,24^ impact on TP53 function. The other *Trp53* mutation (c.157dupT) was a frameshift insertion in exon 4 in the linker domain and the human equivalent (W53Cfs*4) is predicted to have a high impact on TP53 function (Ensembl Variant Effect Predictor). Both human mutations were found in the COSMIC database of mutations, however, none of the observed variants in either gene were specific for poorly differentiated or NEPC tumors in mice irrespective of genotype.

A previously described NEPC score estimates the likelihood a test sample is clinical NEPC based on a set of 70 differentially expressed NEPC-related genes^2^. We calculated the NEPC score for each tumor focus and found that poorly differentiated tumor foci had significantly higher NEPC scores compared to conventional adenocarcinoma and were similar to values obtained from a cohort of patient NEPC tumors (Fig. 2b). Interestingly, tumor foci with divergent differentiation (squamous, poor, osteoid) from castrated mice also had NEPC scores similar to clinical NEPC and were significantly higher than adenocarcinoma of intact tumors (*p* = 0.0004). This suggests that castration results in the activation of an NEPC molecular program regardless of histology.

We then compared the expression differences between distinct histological tumor foci to identify differentially expressed pathways associated with accelerated tumor progression. Ward’s hierarchical clustering was performed using a 2,043 gene signature that distinguished *PRN* poorly differentiated/NEPC foci from *PN* adenocarcinoma foci (adj. *p* < 0.05, |(Log_2_ (Fold Change)| >2), which revealed three distinct sets of gene clusters (Fig. 2c). Genes found in clusters 1 and 2 were highly upregulated in poorly differentiated and NEPC-like samples and were enriched with targets of E2F, MYC, and PRC2 complexes, as well as cell cycle, neural lineage, and EMT genes (Fig. 2d, e). Adenocarcinoma foci were enriched with EMT genes, PRC2 complex target genes, and AR signaling/prostate developmental genes (Supplementary Fig. 3b, c). Interestingly, we observed an enrichment of genes associated with a loss of TP53 signaling in poorly differentiated tumor foci compared to adenocarcinoma despite no distinguishing loss of function mutations or change in *Trp53* expression (Supplementary Fig. 3a, c, d).

### Single cell-based approaches reveal NEPC-like subpopulations

**Fig. 3:**
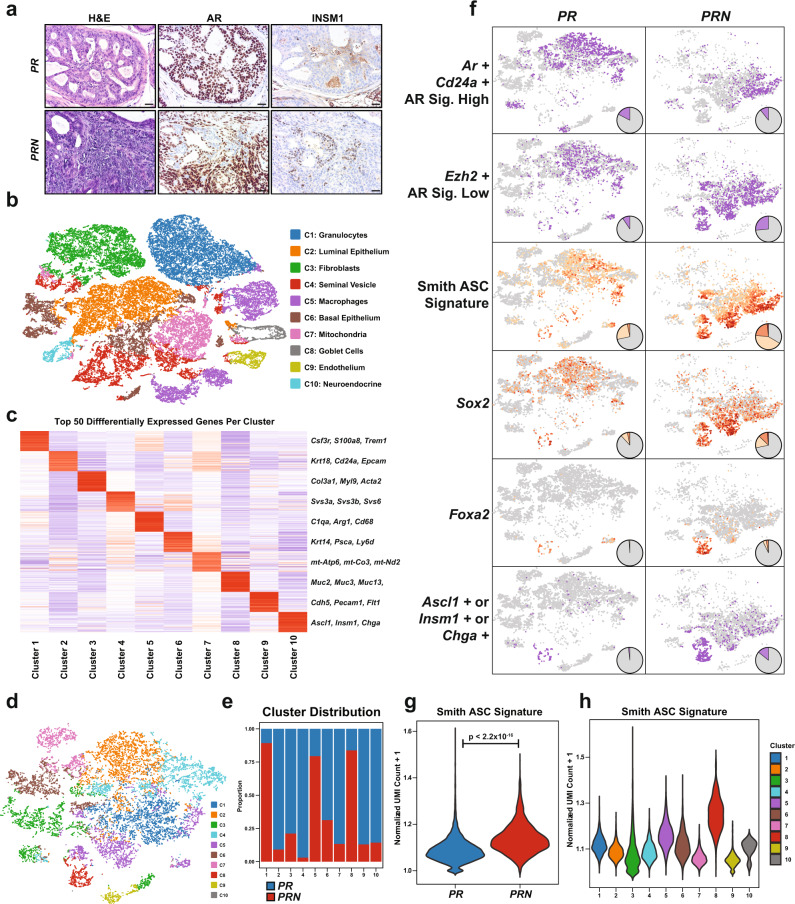
Single cell-based approaches reveal NEPC-like subpopulations. **a** H&E and IHC staining of AR and INSM1 in samples used for scRNA-seq. Scale bar: 50 μm. **b** The combined t-SNE plot of scRNA-seq data from intact *PR* (*n* = 3) and *PRN* (*n* = 3) mice. **c** Clustering of the top 50 most differentially expressed genes between clusters. **d** Reclustering of combined luminal, basal, and neuroendocrine populations from *PR* (*n* = 3) and *PRN* (*n* = 3) mice. **e** Distribution of genotypes between clusters identified in panel **d**. **f** Expression of indicated markers or signature in *PR* (*n* = 3) compared to *PRN* (*n* = 3) mice. Pie charts show proportions of positive vs. negative cells or high/medium/low/negative gene expression. **g** Violin plot of Smith ASC score^25^. **h** ASC score by cluster from panel **d**.

We next sought to characterize the temporal evolution of tumors in *PR* and *PRN* mice. To this end, we performed IHC staining on prostate tissues from 8-week old intact mice and found that while prostates from *PR* mice were composed of AR-positive, HGPIN lesions, tumors from *PRN* mice contained large regions of AR-positive adenocarcinoma, as well as neighboring foci comprised of AR-negative, INSM1-positive cells (Fig. 3a). In order to better understand this heterogeneity we observed in the developing tumors, we performed single-cell RNA sequencing (scRNA-seq) on prostates collected from 8-week old *PRN* mice (*n* = 3), as well as age-matched *PR* mice (*n* = 3) and sought to identify subpopulations that may contribute to tumorigenesis. K-means clustering and UMAP projection of the combined data across both genotypes revealed 10 major cell clusters of gene expression (Fig. 3b). Differential expression analysis between clusters produced a list of the top 50 most differentially expressed genes by cluster (Fig. 3c and Supplementary Table 2), and we assigned identities to each cluster using the expression of known markers (Fig. 3b). We restricted further analyses to include populations that were identified as luminal epithelium, basal epithelium, or neuroendocrine cells on the basis of *Krt5, Krt8, Cd24a, Trp63*, and *Insm1* expression. Re-clustering of this subset allowed for further delineation of the heterogeneity within these populations (*PR* mice: 8,151 cells; *PRN* mice: 5,160 cells) (Fig. 3d). We then assessed what proportion of each cluster was comprised of cells from each genotype and found striking differences in composition (Fig. 3e). For example, we found that 75% of the cells in cluster C8, which corresponds to the neuroendocrine population, were from *PRN* tumors compared to 25% from *PR* tumors. Using gene expression, we identified a population of cells in *PR* tumors that were positive for the luminal markers *Ar* and *Cd24a,* as well as had high levels of AR signaling activity assessed by *Fkbp5* expression (17.3% of the total cells) (Fig. 3f). This population was reduced nearly 2-fold in *PRN* tumors (10.6% of the total cells) and instead, there was a nearly 3-fold expansion of cells that expressed *Ezh2* and had low levels of AR signaling activity (26.5% in *PRN* vs. 9.3% in *PR*), suggesting a transition away from an AR-dependent state (Fig. 3f). GSEA performed on the differentially expressed genes between the *Ezh2* + /AR signaling low and *Ar* + */Cd24a* + populations revealed enrichment for known PRC2, EED, and SUZ12 targets (Supplementary Fig. 3e). These data are consistent with previous findings that N-Myc and EZH2 cooperate to suppress AR signaling^6,7^. Furthermore, the *Ezh2*-positive/AR-signaling low population also had increased expression of a previously published adult stem cell (ASC) signature score^25^ and expression of *Sox2*, consistent with the transition away from an adenocarcinoma state (Fig. 3f). Interestingly, we also identified a population of cells that expressed a number of known neuroendocrine markers, including *Ascl1*, *Chga*, *Foxa2*, and *Insm1* (Fig. 3f). The ASC signature score was significantly higher in *PRN* tumors compared to *PR* tumors (Fig. 3g) and was found to be enriched in the cluster containing neuroendocrine cells (Fig. 3h).

### Cell trajectories towards a neuroendocrine-like lineage

**Fig. 4:**
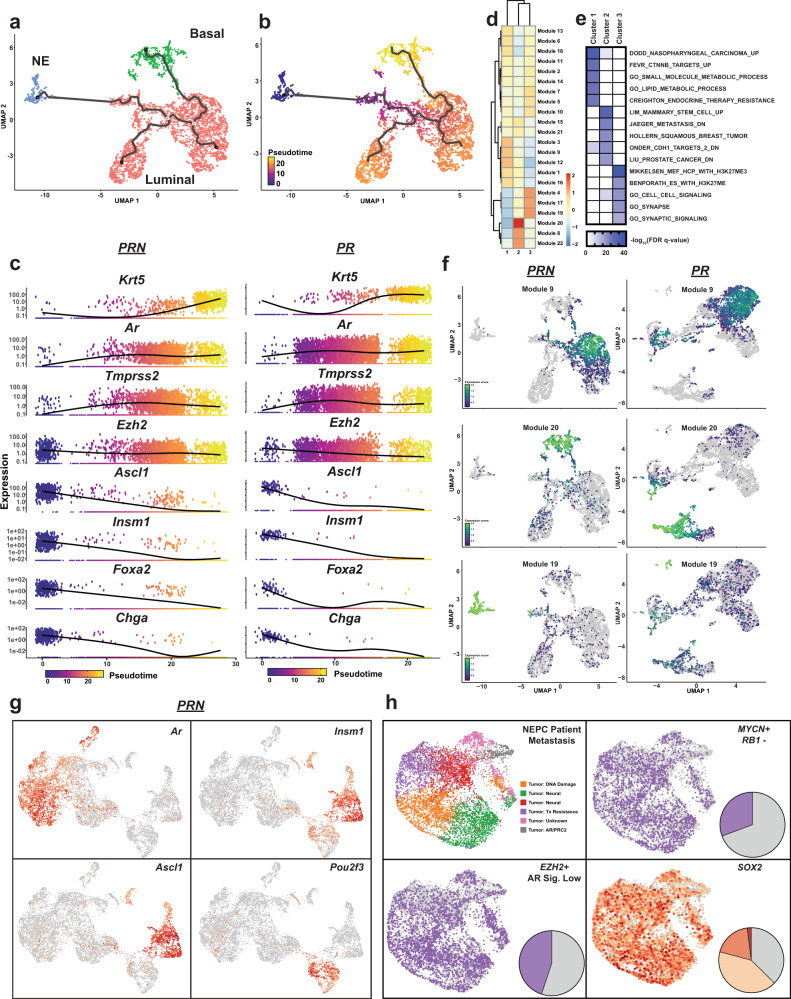
Cell trajectories towards a neuroendocrine-like lineage. **a** UMAP projection of luminal (red), basal (green), and neuroendocrine (blue) populations from *PRN* mice (*n* = 3). **b** Pseudotime trajectories projected onto UMAP from panel **a**. Color reflects pseudotime distance (neuroendocrine population located at *t* = 0). **c** Gene expression levels of indicated genes in individual cells over pseudotime in *PRN* (left) and *PR* (right) mice. Color reflects pseudotime distance (neuroendocrine population located at *t* = 0). **d** Heatmap and clustering of gene expression modules which vary significantly as a function of pseudotime in *PRN* mice. **e** GSEA results showing the top 5 gene sets enriched in modules 9, 19, and 20. **f** Expression of genes from modules 9, 19, and 20 projected onto *PRN* (left) and *PR* (right) mice. **g** Expression of indicated genes in luminal and neuroendocrine cells of 6-week-old (*n* = 2) and 8-week-old (*n* = 3) *PRN* mice. **h** UMAP of scRNA-seq data from a metastatic NEPC sample. Clusters were named based on GSEA results of differentially expressed genes between clusters.

In order to understand the transcriptional relationships between the epithelial and neuroendocrine cell populations, we employed a computational method^26^ to define cell trajectories between the given lineage states. Clustering of the cells derived from *PRN* tumors alone again revealed three distinct cell populations consisting of the basal and luminal epithelium, as well as a neuroendocrine population on the basis of *Krt5, Krt8, Cd24a, Trp63*, and *Insm1* expression (Fig. 4a). Cell trajectories and UMAP projections highlighted the existence of a transition between luminal epithelial cells and neuroendocrine cells (Fig. 4b). This was further confirmed by assessing the expression of known marker genes as a function of pseudotime distance from the neuroendocrine population. Keratin 5 (*Krt5*), a known basal epithelial cell marker, was highly expressed in cells with the greatest pseudotime distance from the neuroendocrine population and was largely restricted to the basal epithelial cell population (Fig. 4c). Levels of *Ar* and its target gene *Tmprss2* were highly expressed in luminal and basal populations and showed low levels of expression in the neuroendocrine population (Fig. 4c). Robust levels of *Ezh2* were observed throughout all cells, with a modest increase of expression in the neuroendocrine population (Fig. 4c). As expected, neuroendocrine markers such as *Ascl1*, *Insm1*, *Foxa2*, and *Chga* were all highly expressed in the neuroendocrine population (Fig. 4c). Surprisingly, this analysis revealed small populations of cells in the luminal epithelial cluster which expressed low levels of these neuroendocrine markers, despite being a significant pseudotime distance away from the neuroendocrine population (Fig. 4c). While the terminal neuroendocrine state also existed in *PR* tumors, the appearance of these early-transition cells occurred with higher frequency and at an earlier point in pseudotime in *PRN* tumors and suggests that this approach can potentially identify cells early in a transition to a neuroendocrine state (Fig. 4c). We then took an unbiased approach to identify gene modules that were differentially expressed as a function of pseudotime. We identified 22 modules whose expression levels correlated with the three cell states (Fig. 4d). GSEA performed on the genes from the most highly expressed module in each cluster revealed differences between the clusters, most notably including enrichment of EZH2-related developmental and neural gene sets in the neuroendocrine cluster (cluster 3, Fig. 4e). The projection of the expression score of the three most highly expressed modules corresponded, as expected, to the three cell states on which they were defined (Fig. 4f). Intriguingly, while modules 9 and 20 were fairly restricted to luminal and basal populations respectively, module 19 was highly expressed in the neuroendocrine cells, as well as a subset of luminal cells that were closest to the neuroendocrine population on the computed cell trajectory (Fig. 4f). The corresponding module in *PR* tumors, while able to identify the neuroendocrine population, did not show a marked enrichment in the cells contained in the luminal population that were along the computed cell trajectory (Fig. 4f and Supplementary Fig. 4a–d).

To better understand the evolution of the cells expressing neuroendocrine markers, we performed scRNA-seq at an earlier time point (6 weeks of age, *n* = 2). At this age, prostates of *PRN* mice contained primarily HGPIN, as well as foci of AR-/INSM1- invasive carcinoma (Supplementary Fig. 4e). Clustering analysis of all cells in the prostate revealed distinct neuroendocrine and luminal populations (Supplementary Fig. 5). A subsequent analysis based on the combined data in luminal and neuroendocrine cells from the 6-week and 8-week time points uncovered two distinct clusters of cells that were AR negative and displayed intermediate to high levels of neuroendocrine marker gene expression (*Insm1*), as well as ubiquitin C-terminal hydrolase L1 (*Uchl1*) (Fig. 4g and Supplementary Fig. 4f). Moreover, these two populations were distinguished by differential expression of *Ascl1/Chga* vs. the Oct11-encoding gene *Pou2f3* (Fig. 4g, Supplementary Fig. 4f, and Supplementary Fig. 5). These populations were observed in both 6-week and 8-week time points and the cells from both timepoints contributed to all clusters (Supplementary Fig. 4g). We next sought to identify genes that marked the intermediate population of cells that were potentially transitioning towards a neuroendocrine phenotype. We performed a differential expression analysis and identified 1,822 genes that were significantly enriched in the cluster of cells found between the luminal and neuroendocrine populations, including *Pou2f3*, *Ovol3*, and *Ascl2* (Fig. 4g, Supplementary Table 3, and Supplementary Data 1).

To determine the clinical relevance of these findings, we performed similar scRNA-seq based approaches on a clinical sample isolated from a patient at New York-Presbyterian Hospital/Weill Cornell Medicine who presented with AR-positive prostate adenocarcinoma and AR-negative NEPC liver metastasis. Genomic profiling of the primary tumor identified a 5.8 Mb focal deletion of *PTEN* (10:89,536,129-90,122,394) and a missense mutation in *TP53* (R273C). While the NEPC liver metastasis shared the *PTEN* deletion with the primary tumor and also harbored a missense mutation in *TP53* (R114C), the metastatic lesion carried a nonsense mutation and predicted loss-of-function of *RB1* (R579X) (Supplementary Table 4). The patient progressed following androgen deprivation therapy, docetaxel, and carboplatin treatment and developed an NEPC pleural effusion that was isolated and subjected to scRNA-seq. Analysis of the scRNA-seq data revealed numerous subclusters within the main tumor cell population after removal of hematopoietic and stromal populations (Fig. 4h). Gene Ontology analysis of the differentially expressed genes between the clusters revealed populations that were enriched for varying cellular processes, including DNA damage response and neural lineage pathways. Similar to the genetic context of the mouse models, the NEPC patient sample contained a substantial number of *MYCN*-positive/*RB1*-negative cells (Fig. 4h). We also observed a high number of *EZH2* + /AR signaling low cells (Fig. 4h), many of which also expressed *SOX2* (Fig. 4h) and *UCHL1* (Supplementary Fig. 4h), consistent with our findings in *PRN* mice. Finally, application of the ASC signature score^25^ revealed a strong overlap with cells positive for the marker *MKI67*, indicating the enhanced proliferative capacity of this population (Supplementary Fig. 4h). We also performed scRNA-seq on a second prostate cancer sample from a patient who developed a local recurrence in the prostate with mixed small cell carcinoma and adenocarcinoma following ADT treatment. Consistent with our findings in *PRN* mice, we found a cluster of cells that were strongly positive for the neuroendocrine marker genes *INSM1* and *CHGA*, as well as *SOX2* and *UCHL1* (Supplementary Fig. 4i) in addition to a large cluster of cells which showed markedly reduced expression of these genes. RNA velocity analysis of these two populations revealed the existence of cell trajectories which would suggest that cells would most likely transition towards the neuroendocrine cluster (Supplementary Fig. 4j).

### Distinct chromatin accessibility patterns exist in neuroendocrine subpopulations

**Fig. 5:**
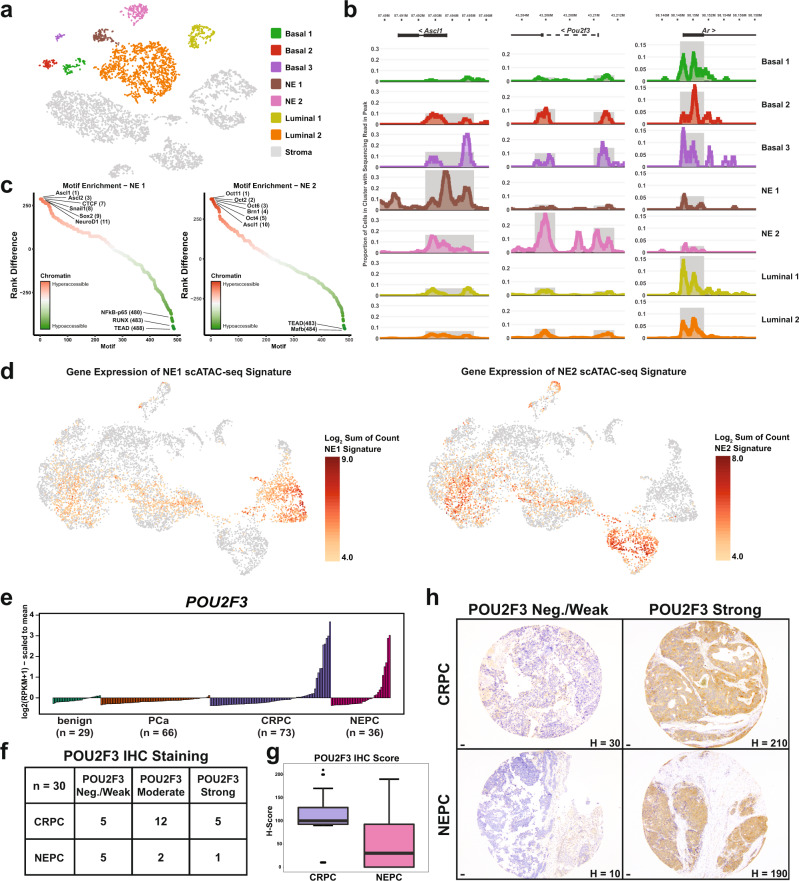
Distinct chromatin accessibility patterns exist in neuroendocrine subpopulations. **a** t-SNE plot of scATAC-seq data from *PRN* mice. **b** Chromatin accessibility in the subpopulations at the indicated loci. **c** Graphs of differential transcription factor motif enrichment in the indicated NE subpopulations called on regions of hyper-accessible and hypo-accessible chromatin between clusters. **d** Gene expression levels of the top 50 most differentially accessible genes in the NE1 and NE2 populations. Genes identified in Supplementary Table 5 and projected onto UMAP from Fig. 4g. **e** *POU2F3* expression in benign (*n* = 29), locally advanced prostate cancer (PCa) (*n* = 66), CRPC (*n* = 73), and NEPC (*n* = 36) patient samples. **f** Summary of POU2F3 IHC staining on clinical tissue microarray. **g** Quantification of IHC scores in TMA by patient diagnosis (CRPC: *n* = 22; NEPC: *n* = 8). Box represents 25%−75% percentile with median denoted as a line. Whiskers extend +/−1.5× interquartile range. Data points that lie outside the whiskers are plotted individually. **h** Representative images of POU2F3-strong and POU2F3-negative/weak patient samples. Scale bar: 50 μm.

Based on the transcriptional heterogeneity observed during tumorigenesis and the existence of cell trajectories that suggested lineage plasticity between luminal epithelium and neuroendocrine cells, we sought to identify the factors that may be responsible for promoting this change in lineage state. To this end, we performed a single-cell assay for transposase-accessible chromatin followed by sequencing (scATAC-seq) from an 8-week old *PRN* mouse to determine regions of differential chromatin accessibility between populations. Following library generation and sequencing, fragment sizes varied with a periodicity roughly corresponding to nucleosome spacing (Supplementary Fig. 6a). As expected, regions with the highest levels of accessibility were centered around the transcription start site (TSS) of genes (Supplementary Fig. 6b). After clustering the chromatin accessibility data, we assessed accessibility at a number of lineage-specific loci (such as *Krt8*, *Psca*, *Ar, Insm1*, *Ptprc*, *Col3a1*, and *Pecam1*) to assign identities (luminal, basal, neuroendocrine, stroma) to the cells (Supplementary Fig. 6e–g). Moreover, we observed distinct subclusters among the basal, luminal, and neuroendocrine populations (Fig. 5a). The two neuroendocrine subclusters demonstrated high accessibility at known neuroendocrine marker gene loci, therefore we performed a differential accessibility analysis between the NE1 and NE2 populations to uncover differences between these two clusters (Supplementary Table 5 and Supplementary Data 2). From this comparison, we found that the NE1 population had enrichment of chromatin accessibility at the *Ascl1* and *Foxa2* loci while the NE2 population had a significant enrichment at the *Ascl2* and *Pou2f3* loci (Fig. 5b, Supplementary Fig. 6g, and Supplementary Table 5), similar to the scRNA-seq data (Fig. 4g). In contrast, the genomic loci for *Ar* and the AR target gene *Tmprss2* showed high levels of accessibility in all luminal and basal populations and virtually no accessibility in either of the neuroendocrine populations (Fig. 5b and Supplementary Fig. 6c). We identified regions of chromatin with differential accessibility between all the subpopulations and performed motif analyses to determine the transcription factor binding sites which were enriched in the hyper-accessible and hypo-accessible regions. As expected, luminal populations were enriched for GATA motifs along with glucocorticoid response elements (GRE) and androgen response elements (ARE) (Supplementary Fig. 6d). Interestingly, the neuroendocrine populations were characterized by differential enrichment of ASCL1 motifs in the NE1 population and of OCT/POU family members in the NE2 population (Fig. 5c), consistent with differences seen in accessibility at the *Pou2f3* loci (Fig. 5b and Supplementary Table 5). To better appreciate the emergence of the two neuroendocrine populations, we also performed scATAC-seq in prostates collected from 6-week old *PRN* mice (*n* = 2) and combined the data with the 8-week time point. Similar to the 8-week only data, the combined data again revealed two populations with high chromatin accessibility at the *Insm1* locus but with differential accessibility at the *Ascl1* and *Pou2f3* loci (Supplementary Fig. 6h, j). Moreover, these populations were comprised of cells from both the 6-week and 8-week time points (Supplementary Fig. 6i). In agreement with our scRNA-seq findings, these data suggest the existence of discrete neuroendocrine populations which may be driven by distinct transcriptional programs. To determine the relationship between the NE subpopulations observed by scRNA-seq and scATAC-seq, we created a gene signature from the 50 most differentially accessible loci in the NE1 and NE2 scATAC-seq populations. We then assessed the expression of these genes in the scRNA-seq data and found that the NE1 scATAC-seq signature was enriched in the *Ascl1*-positive scRNA-seq subpopulation while the NE2 scATAC-seq signature was enriched in the *Pou2f3*-positive scRNA-seq subpopulation (Fig. 5d). Finally, we investigated the expression levels of *POU2F3* in prostate cancer patients. RNA-seq data from a clinical cohort revealed *POU2F3* is expressed in a subset of CRPC and NEPC patients (Fig. 5e). Similar to the observations made in the *PRN* mice, expression of *POU2F3* appeared to be mutually exclusive with *ASCL1* (Supplementary Fig. 7a) and was inversely correlated with *RB1* expression (Supplementary Fig. 7b, d). Moreover, *POU2F3* expression was positively correlated with the neuroendocrine marker *ENO2* (Supplementary Fig. 7c). To validate the expression of POU2F3 at the protein level, we performed POU2F3 immunohistochemistry on a tissue microarray (TMA) of prostate cancer cases containing evaluable material for 22 CRPC patients and 8 NEPC patients. A blinded pathologist review revealed 77.3% of the CRPC cases and 37.5% of the NEPC cases had moderate to strong POU2F3 staining (Fig. 5f–h). These data demonstrate that POU2F3 is expressed in clinical CRPC and NEPC cases and may serve as a biomarker of CRPC to NEPC transition. Further work is needed, however, to understand the heterogeneity in POU2F3 expression within the CRPC and NEPC populations.

### The N-Myc cistrome is altered upon *Rb1* Loss

**Fig. 6:**
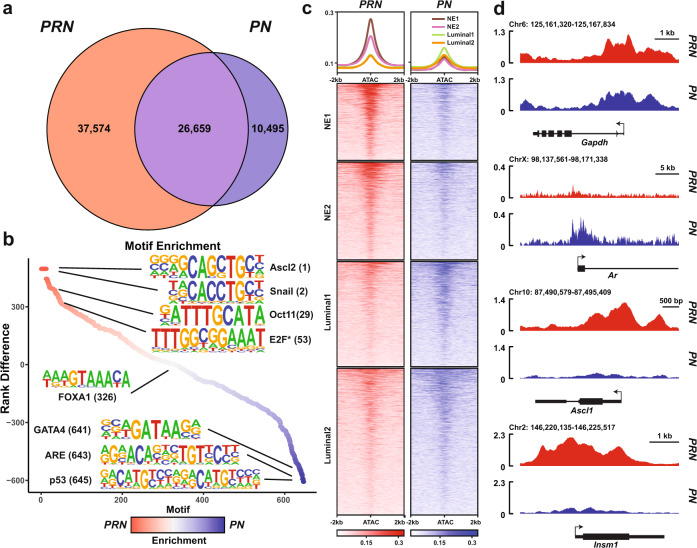
The N-Myc cistrome is altered upon Rb1 loss. **a** Venn diagram showing the overlap of N-Myc binding by ChIP-seq from *PRN* (*n* = 3) and *PN* (*n* = 2) tumors (peak cutoff: *q* < 0.0001). **b** Differential transcription factor motif enrichment between N-Myc binding sites in *PRN* and *PN* tumors. **c** Heatmaps of N-Myc binding in *PRN* and *PN* tumors at regions of accessible chromatin associated with neuroendocrine and luminal subpopulations by scATAC-seq in Fig. 5a. **d** N-Myc binding at indicated loci in *PRN* (*n* = 3) and *PN* (*n* = 2) tumors.

Based on single-cell-based sequencing data from the GEMs, we observed that the overexpression of *MYCN* in combination with the loss of *Rb1* resulted in the rapid formation of aggressive, metastatic tumors with neuroendocrine-like features. Thus, we hypothesized that the loss of *Rb1* would synergize with *MYCN* overexpression to reshape the N-Myc cistrome and drive tumor progression. To address this, we performed N-Myc chromatin immunoprecipitation sequencing (ChIP-seq) on multiple, independent tumors collected from *PN* or *PRN* mice. Consistent with its role as a transcription factor, N-Myc binding was highly enriched near the transcription start site (TSS) of genes in both genotypes (Supplementary Fig. 8a). We found 36,789 N-Myc peaks in *PN* tumors, in relative agreement with our previous studies of N-Myc binding in human prostate cancer cells^6^. Surprisingly, following the loss of *Rb1*, the N-Myc cistrome was dramatically expanded to 62,171 binding sites in *PRN* mice with nearly 60% of these sites corresponding to new binding sites not seen in *PN* mice alone (Fig. 6a). In addition, GSEA of the *PN* and *PRN* unique peaks revealed distinct sets of genes bound by N-Myc between the conditions. While many luminal epithelial and hormone-responsive gene sets were enriched in *PN* tumors, a large enrichment of PRC2 complex targets, epigenetically marked developmental, and neural lineage gene sets were found in *PRN* tumors (Supplementary Fig. 8b). To determine the transcriptional co-factors which may contribute to this differentially remodeled cistrome, we performed motif enrichment analysis between N-Myc peaks unique to the *PN* and *PRN* mice. While the most differentially enriched motifs in *PN* tumors consisted of TP53 and ARE motifs, *PRN* tumors were enriched for ASCL, POU2F3 (OCT11), and SNAIL family motifs (Fig. 6b). More strikingly, a high degree of enrichment was observed for a motif consistent with the E2F family of transcription factors^27^ (Fig. 6b). To assess the degree of altered N-Myc binding and chromatin accessibility, we integrated the scATAC-seq and the ChIP-seq data and restricted the analysis to regions of differential accessibility between subpopulations. In the presence of wild-type levels of *Rb1*, N-Myc was bound at regions of accessible chromatin associated with luminal populations (Fig. 6c). In the context of *Rb1* loss, N-Myc binding was reduced in the luminal-specific regions and instead was dramatically enriched in regions that were accessible only in the neuroendocrine populations (Fig. 6c). Specifically, we found that N-Myc binding was reduced at the *Ar* locus and redirected to neuroendocrine-associated genes, such as *Ascl1* and *Insm1*, in *PRN* tumors (Fig. 6d). Importantly, the loss of *Rb1* appeared to remodel the N-Myc cistrome in a very specific manner, as binding at the *Gapdh* locus (a known N-Myc target gene) remained consistent between *PN* and *PRN* tumors (Fig. 6d). Together, these data suggest that the loss of *Rb1* synergizes with N-Myc overexpression to redirect the N-Myc cistrome to new genomic loci to regulate gene expression, possibly with the help of ASCL-family and E2F-family members.

### N-Myc and Rb1 loss tumors adopt NEPC methylation programs

**Fig. 7:**
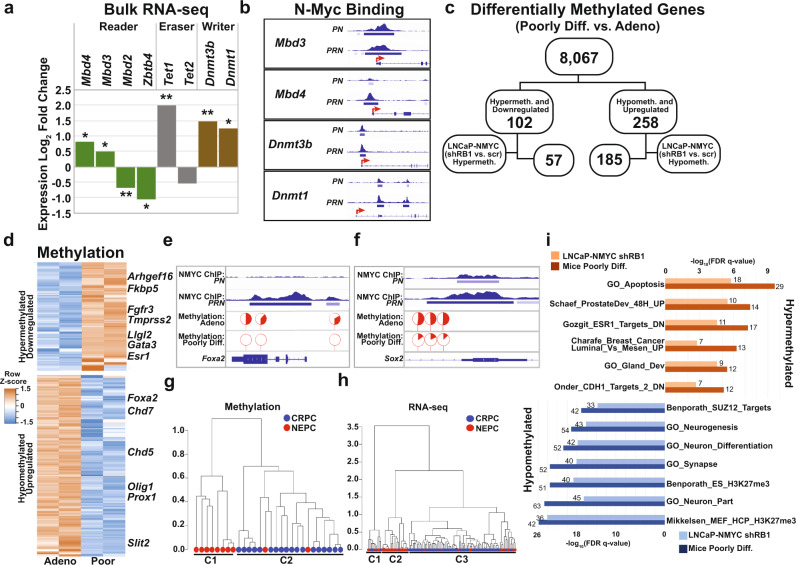
N-Myc and Rb1 loss tumors adopt NEPC methylation programs. **a** Log_2_ fold change in expression of DNA methylation regulators which were significantly deregulated in poorly differentiated GEM tumors. Benjamini-Hochberg adjusted two-sided *t*-test *p < 0.05, **p < 0.01 (*p* = 0.0437, 0.0267, 0.0089, 0.0164, 0.0031, 0.0582, 0.0013, 0.0131). **b** Normalized N-Myc binding peaks from ChIP-seq in poorly differentiated and adenocarcinoma tumors near the transcription start site of each gene. **c** Flowchart of the downstream analysis with differentially methylated genes of poorly differentiated tumors compared to adenocarcinomas. **d** Heatmap with methylation scores of hypermethylated genes (*n* = 102) and hypomethylated (*n* = 258) in two pairs of tumor tissues. **e**, **f** N-Myc binding and methylation status of upregulated genes *Foxa2* (**e**) or *Sox2* (**f**) in the two histological subtypes of mouse tumors. Red indicates the % of methylated C at each site for each tumor type. **g** Cluster map of CRPC (*n* = 18) and NEPC (*n* = 10) samples using methylation scores of 360 genes (distance method: Correlation, clustering method: Ward D2). *P* values indicated on each of the major clusters are based on two hypergeometric tests for the enrichment of NEPC (*p* = 1.45 × 10^−5^) or CRPC (*p* = 1.45 × 10^−5^) samples in either cluster, respectively. **h** Cluster map of CRPC (*n* = 73) and NEPC (*n* = 36) samples using expression of 360 genes (distance method: Correlation, clustering method: Ward D2). *P* values indicated on each of the major clusters are based on two hypergeometric tests for the enrichment of NEPC (*p* = 1.8 × 10^−10^) or CRPC (*p* = 3.6 × 10^−10^) samples in either cluster, respectively. **i** GSEA results using hypermethylated genes (top panel) from mouse tumors (*n* = 102) and LNCaP cells (*n* = 57) and hypomethylated genes (bottom panel) from mouse tumors (*n* = 258) and LNCaP cells (*n* = 185) (bottom panel). The number on each horizontal bar indicates the number of genes overlapping with the respective gene set.

Differential DNA methylation is another mechanism that may account for expression differences in histologically distinct cancer foci. Based on the bulk RNA-seq data from GEM tumors, we found that DNA methyltransferases (*Dnmt1, Dnmt3b)*, demethylase (*Tet1*), and methyl-CpG binding domain genes *(Mbd3* and *Mbd4)* were differentially upregulated, while *Mbd2* and *Zbtb4* were downregulated in poorly differentiated foci compared to adenocarcinoma foci (Fig. 7a). Similar upregulation of *DNMTs* and *TET1* was observed in NEPC patients compared to CRPC patients^2^. Furthermore, based on the N-Myc ChIP-seq data, we found that N-Myc binding was enhanced at the promoters of *Dnmt1*, *Dnmt3b*, and *Mdb4* in *PRN* (primarily poorly differentiated histology) compared to *PN* tumors (primarily adenocarcinoma histology, Fig. 7b). To determine if these gene regulation differences translated to differential DNA methylation, we performed reduced representation bisulfite sequencing (RRBS). We generated methylation profiles from tissue adjacent to regions that were cored for RNA-seq analysis from mice with poorly differentiated foci or adenocarcinoma foci. Based on these methylation profiles, the adenocarcinoma foci segregated together but distinctly from the poorly differentiated tumors in principal component analysis (Supplementary Fig. 9a). Moreover, the methylation profiles of the same histology were highly correlated (adenocarcinomas, *r* = 0.95; poorly differentiated, *r* = 0.83, Supplementary Fig. 9b) and were consistent with the higher degree of hypomethylation observed in NEPC clinical tumors compared to CRPC tumors^2^. The distribution of differentially methylated sites in the genomic regions and CpG context showed a higher frequency of hypermethylated sites in promoter regions and CpG islands (CpGi) similar to findings from other methylation studies^28^ (Supplementary Fig. 9c). However, hypermethylated promoter sites were enriched at CpGi while hypomethylated promoter sites were preferentially in CpG shores (Supplementary Fig. 9d). Interestingly, hypermethylation in CpGi has previously been reported to be associated with prostate cancer severity^28^. Integration of the RNA-seq data revealed that 102 of the differentially hypermethylated genes were also downregulated in poorly differentiated tumor foci compared to adenocarcinoma foci and were also differentially methylated in clinical NEPC tumors compared to CRPC tumors^2^. Similarly, 258 of the differentially hypomethylated genes in the GEM tumors were also upregulated and differentially methylated in NEPC patient samples (Fig. 7c). To rule out if the difference in methylation was due to different genotypes, the frequency of methylated Cs of all 360 (102 + 258) genes were compared between the pair of poorly differentiated and adenocarcinoma histological subtypes from the same mouse. The majority of these 360 genes had varying methylation levels in the two subtypes which showed concordance with their expression levels (Fig. 7d). Interestingly, *Esr1*, *Fgfr3*, and AR target genes, *Fkbp5* and *Tmprss2*, were differentially hypermethylated and downregulated while regulators of cell differentiation and neural development (e.g., *Slit2, Prox1*, and *Olig1*) were hypomethylated and upregulated in poorly differentiated tumor foci compared to adenocarcinoma foci (Fig. 7d). *Foxa2*, a major regulator of cell fate decision commonly overexpressed in NEPC, was hypomethylated, upregulated, and preferentially bound by N-Myc near its TSS in *PRN* tumors compared to *PN* tumors (Fig. 7e). Unique N-Myc binding sites in *PRN* tumors overlapped with the differentially methylated sites in poorly differentiated tumors compared to adenocarcinoma. *Sox2*, a lineage-defining factor, was found to be within regions of enhanced N-Myc binding in *PRN* tumors with poorly differentiated histology (Fig. 7f).

To address the clinical relevance of methylome data from our mouse model, we used the 360-gene mouse methylation signature to query methylation data from a cohort of 28 well-characterized CRPC (*n* = 18) and NEPC (*n* = 10) samples^2^. Hierarchical clustering segregated the samples into two groups, one of which (cluster 1) was comprised of only NEPC samples (8/8; *p* = 1.4 × 10^−5^, hypergeometric test) while the other (cluster 2) was significantly enriched in CRPC samples (18/20, *p* = 1.4 × 10^−5^, hypergeometric test, Fig. 7g). One of the two NEPC samples that segregated with the CRPC cluster, was also found to group with CRPC when all differentially methylated genes were used for clustering analysis (Supplementary Fig. 9e). The relevance of these 360 genes in NEPC was further investigated by clustering a larger cohort of patient samples (73 CRPC and 36 NEPC) using expression values of the 360 genes. Of the three clusters, one had a mixture of CRPC and NEPC samples, the second was enriched in NEPC (19/20; *p* = 1.8 × 10^−10^, hypergeometric test) and the third was enriched in CRPC (67/79; *p* = 3.6 × 10^−10^, hypergeometric test, Fig. 7h). Similar clustering using the expression of the 360 genes was done in a larger cohort of poorly differentiated and adenocarcinoma mouse tumors, resulting in two clusters, one of which was enriched in adenocarcinoma (5/8) while the other consisted of all poorly differentiated tumors (6/6) (Supplementary Fig. 9f).

To test the robustness of the murine methylation results, we performed RRBS in LNCaP cells engineered to overexpress *MYCN*^5–8^, along with shRNA targeting *RB1*, and compared to control LNCaP cells (Supplementary Fig. 9g). Of the differentially expressed 360 genes, 242 (57 hypermethylated + 185 hypomethylated) were also differentially expressed in LNCaP-NMYC-shRB1 cells compared to LNCaP-NMYC cells engineered with a scrambled shRNA control (Fig. 7c). Cluster analysis of the clinical samples using this 242-gene methylation data segregated NEPC and CRPC samples into discrete subclusters (Supplementary Fig. 9h). We also found that the signaling pathways enriched among the hypermethylated and hypomethylated gene sets in LNCaP cells were similar to what was observed with the 360-gene list (Fig. 7i).

## Discussion

While *RB1* loss is common in prostate cancer and N-Myc overexpression occurs in a large proportion of NEPC, little is known about the potential synergy between these two events. Here, we have used a cohort of CRPC and NEPC patients, exploring both gene expression and outcomes data, to determine that the co-occurrence of *RB1* loss and N-Myc overexpression is associated with worse overall survival than either event alone. To study the mechanism behind this association, we generated a GEM combining dual *Pten* and *Rb1* loss with N-Myc overexpression. These mice rapidly developed tumors with poorly differentiated histology and were significantly more castration-resistant than control animals.

The degree of differences between the transcriptomes of specific histologies (e.g., adenocarcinoma vs. poorly differentiated/NEPC) based on bulk RNA-seq was not surprising given the dramatic phenotypic differences. However, the striking differences in the N-Myc cistrome in the context of *Rb1* loss were intriguing and unexpected, and we hypothesized that cofactors and/or other transcription factors could account for these differences. Interestingly, we observed an enrichment of E2F and MYC targets in poorly differentiated tumor foci compared to adenocarcinoma, suggesting that E2F and N-Myc may have altered or enhanced activity in the transition from adenocarcinoma to a poorly differentiated state. This was also reflected in our ChIP-seq studies where E2F motifs were enriched at N-Myc binding sites that were uniquely found in poorly differentiated tumors. For N-Myc, this is consistent with our previous findings that the N-Myc cistrome can be dynamically regulated^6^. However, in the context of androgen withdrawal, N-Myc binding sites were enriched for Forkhead motifs. Interestingly, the FOXA1 motif was equally enriched in both *PN* and *PRN* tumors. Instead, the enrichment of an E2F motif at N-Myc binding sites in the context of *Rb1* loss suggests that N-Myc binds at E2F target genes and synergizes to drive a tumorigenic transcriptional state. Further work is needed to determine if E2F transcription factors cooperate with N-Myc and redirect N-Myc binding in the context of *RB1* loss. Downregulated genes were enriched in epigenetic reprogramming and PRC2 targets, as well as the epithelial identity, AR signaling, and P53 targets. Our previous work demonstrated that N-Myc activates EZH2 transcriptional reprogramming, which plays an important role in driving neuroendocrine prostate cancer^7^. Enrichment of PRC2 target genes and epigenetically marked genes (H3K27me3, associated with repression) suggests a shift in epigenetic reprogramming mediated by PRC2 when cells transition from adenocarcinoma to a poorly differentiated/NEPC phenotype. We also observed the downregulation of genes related to epithelial identities, such as those that are downregulated by loss of epithelial adhesion molecule E-cadherin (CDH1). Loss of E-cadherin is thought to promote metastasis through the initiation of EMT and invasiveness^29^. We primarily saw metastatic lesions with poorly differentiated histology in our GEMs and mouse allografts, suggesting that poorly differentiated cells have higher metastatic potential due to activation of EMT pathways. Interestingly, our use of organoid-derived subcutaneous allografts revealed that the development of NEPC-like disease can occur in the context of *Rb1* loss and N-Myc overexpression independently of the prostate microenvironment. Further studies are needed to fully appreciate the role of the tumor microenvironment in shaping early prostate tumorigenesis.

Using RRBS to query changes in DNA methylation across our in vivo and in vitro models, as well as patient samples, we found that components of the DNA methylation machinery were deregulated in poorly differentiated/NE tumors compared to adenocarcinomas. This corresponded to differential methylation statuses across a number of clinically relevant loci throughout the genome. Based on our methylation data from GEM tumors, we developed a 360 gene methylation signature that successfully segregated clinical CRPC and NEPC samples. It remains to be seen how levels of DNA methylation track with levels of H3K27me3 and chromatin accessibility.

Given the heterogeneity in the developing tumors, single-cell-based approaches allowed for a refined characterization of the oncogenic transformation. Single-cell RNA-seq studies recapitulated our previous reports of the role of N-Myc and EZH2 cooperating to suppress AR signaling^6,7^. We have also demonstrated that the population of cells expressing known neuroendocrine markers also showed increased expression of a gene expression signature correlated with poor survival in patients^25^. Of particular note, we found an upregulation of a number of neuroendocrine marker genes in cells that were not part of the distinct neuroendocrine cluster and were a large distance away in pseudotime. These data strongly support the notion that cells transition from a luminal-like state towards an NEPC-like state and are not simply an expansion of existing neuroendocrine precursor cells. Moreover, as many of these cells expressed AR but had low levels of AR target genes, it suggests that pharmacologic inhibition of EZH2 may restore androgen signaling and reverse the induced NE transcriptional program. This is particularly relevant as a number of clinical trials using EZH2 inhibitors are currently in progress, including many in prostate cancer (NCT03480646, NCT04179864, NCT03460977). In addition, both our scRNA-seq and scATAC-seq studies found two distinct neuroendocrine clusters on the basis of transcription and chromatin accessibility. These two clusters differed in their expression of *Ascl1 and Pou2f3*, as well as in their enrichment for ASCL and POU/OCT transcription factor family binding motifs. Recent work in small cell lung cancer (SCLC) has found Notch activation in c-Myc-driven tumors can promote a switch between ASCL1-positive, NEUROD1-positive, and YAP1-positive states^30^. Our previous studies have shown that, while c-Myc and N-Myc share a number of common target genes, the N-Myc cistrome in prostate cells encompasses a larger and more diverse set of transcriptional targets^6^. We did find enrichment of NeuroD1 motifs in the scATAC-seq data from one of the neuroendocrine subpopulations. It is possible that changes to accessible motifs mark populations that are undergoing a transition between states. Additional work is needed to understand how these two subpopulations are regulated and if there are different lineage outcomes for a cell depending on which of the populations served as a cluster of origin. Moreover, data from clinical samples also reveal *POU2F3* is expressed in a subset of CRPC and NEPC patients, and more mechanistic insight is needed to determine the function of *POU2F3* during disease progression.

Altogether, our studies have identified a population of cells that downregulate AR signaling and undergo a lineage transformation to a neuroendocrine-like phenotype following N-Myc over-expression and *Pten/Rb1* co-loss. This lineage change was accompanied by changes in chromatin accessibility and N-Myc binding at a number of clinically relevant genes. Finally, we identified a methylation signature that may inform future clinical trials using EZH2 inhibitors as to which patients are most likely to benefit from EZH2 inhibition and as a biomarker to identify patients who show a beneficial response. These studies have shed light on the molecular mechanisms by which N-Myc overexpression and *Rb1* loss synergize to drive the development of neuroendocrine prostate cancer and have provided new avenues of exploration towards identifying and validating new therapeutic targets.

## Methods

### Animals

Mice were maintained and all procedures were performed on male mice following protocols approved by the WCM-IACUC (protocol no. 2008-0019). Mice carrying the CAG-LSL-MYCN human transgene at the *Rosa26* locus (LSL-MYCN^+/+^)^31^ were crossed with mice expressing Cre recombinase under the control of rat *Probasin*, a prostate-specific promoter (Jackson Laboratory, #026662, RRID:IMSR_JAX:026662, along with *Pten* homozygous floxed alleles (*PbCre*^*+/−*^*; Pten*^*f/f*^). Resulting males that carried the *MYCN* transgene, Cre recombinase, and *Pten* floxed alleles were crossed with females carrying *Rb1* homozygous floxed alleles (Jackson Laboratory, #026563, RRID:IMSR_JAX:026563). Prostate-specific Cre expression results in removal of LSL cassette by Cre and human N-Myc expression driven by the chicken actin promoter. Simultaneously, Cre recombinase converts the *Pten* and *Rb1* floxed alleles to knockout alleles in the mouse prostate. All lines of mice were bred on the same mixed genetic background (C57BL6/129 × 1/SvJ) and have been previously described^7^.

### Human subjects

Male patients were enrolled on an Institutional Review Board (IRB)-approved protocol with informed consent (WCM IRB no. 0905010441, 1305013903, and 1210013164). For these studies, 76 metastatic castrate-resistant prostate adenocarcinoma (*n* = 55) and neuroendocrine prostate cancer (*n* = 21) with survival data were selected for further analysis. Inclusion criteria and additional information on this patient cohort have been previously described^2^.

### Human prostate cancer cell line culture

Human LNCaP male prostate cancer cell lines were obtained from ATCC and maintained as recommended. Cell line authentication was performed using short tandem repeat (STR) DNA analysis. LNCaP prostate cancer cell lines overexpressing N-Myc or empty vector control were cultured in RPMI 1640 medium (Gibco, 11875-093) supplemented with 10% FBS (Gemini, 900–108) and 1% penicillin/streptomycin (Gibco, 15140-122).

### Mouse 3D organoid cell culture

Mouse prostate and tumor-derived organoid 3D cultures were maintained using a protocol adapted from a previously published method^32^. Briefly, prostates were dissected from mice approximately 7–8 weeks of age. Murine prostates were minced and enzymatically digested using collagenase type II (Gibco, 17101-015), resuspended at 5 mg/mL in R++ media (Advanced DMEM/F12/2 mM GlutaMAX/10 mM HEPES) at 37 °C for 1 h. Digested tissue was washed once in R++ media, resuspended in 5 mL TrypLE Express (Gibco, 12604-021), and further digested at 37 °C for 15 min. Cells were washed once in mouse organoid media (see Supplementary Table 6) and resuspended in 90% growth factor reduced Matrigel (Corning, 354230) diluted in mouse organoid media. Each mouse prostate was resuspended in approximately 1 mL of Matrigel mixture. Matrigel and cells were plated as droplets onto a 10 cm cell culture dish and placed in a CO_2_ incubator (5% CO_2_, 37 °C) for 15 min to allow Matrigel to solidify. 10 mL of prewarmed 37 °C mouse organoid media was gently added to cells and cells were maintained in a CO_2_ incubator (5% CO_2_, 37 °C). The media was changed every 3 days. Organoids were split weekly 1:3. Matrigel and organoids were dissolved in cold R++ media, spun down at 750 x *g* for 5 min at 4 °C, and resuspended in 5 mL of TrypLE Express. Matrigel and organoids were digested at 37 °C for 15 min, washed in mouse organoid media, and re-plated in 90% Matrigel and mouse organoid media.

### Adenoviral transduction of mouse organoids

Three-dimensional (3D) mouse organoids were created from PbCre negative mice with floxed alleles (*Pten*^*f/f*^, *Pten*^*f/f*^*; LSL-MYCN*+, *Pten*^*f/f*^*; Rb1*^*f/f*^, *Pten*^*f/f*^*; LSL-MYCN*+*; Rb1*^*f/f*^) as described above. Newly created 3D organoids were passaged three times before being plated in 2D cell culture. Organoids were dissociated as described above and dissociated cells were plated on 1% Collagen I Rat Protein (Gibco, A1048301) coated cell culture flasks. After an additional two passages, cells were seeded at 250,000 cells/well in a 6-well plate. The following day, cells were infected at an MOI of 500. We used recombinant empty vector (Vector Biolabs, 1660) as a control and Cre-expressing (Vector Biolabs, 1774) human adenoviruses type 5 expressing Red Fluorescent Protein (RFP) and a Cre recombinase under the control of the CMV promoter. The following day, cells were washed once in PBS and new media was added. Cells were propagated for an additional few passages before growing cells as 3D organoids. The resulting *P*, *PN*, and *PRN* organoids were named according to the adenovirus used (e.g., the empty vector: *P*^*Con*^, *PN*^*Con*^, *PRN*^*Con*^; or Cre-expressing adenovirus: *P*^*Cre*^, *PN*^*Cre*^, *PRN*^*Cre*^).

### Allografts

Approximately 1 × 10^6^ cells resuspended in 100 uL PBS were diluted 1:1 in Matrigel (Corning, 354230) and injected subcutaneously into the flanks of nude (NU/J) mice (Jackson Laboratories, #002019, RRID:IMSR_JAX:002019). Allograft growth was tracked over time using calipers for volume measurements and mice were luciferase imaged following intraperitoneal injection of 150 mg/kg luciferin (Gold Biotechnology, LUCK-1G) using the IVIS Spectrum In Vivo Imaging System.

### Immunoblot analysis

Protein lysates were collected in RIPA buffer (Thermo Scientific, 89901) supplemented with protease inhibitor cocktail and phosphatase inhibitors (Thermo Scientific, 78428/78430). The protein concentration of each sample was determined using the DC Protein Assay Kit (Bio-Rad, 5000116). Protein samples (30 μg) were resolved by SDS–PAGE (Bio-Rad, 4561084) and transferred onto a PVDF membrane (Thermo Scientific, IB24002). Membranes were blocked in 5% non-fat milk (Bio-Rad, 1706404) or bovine serum albumin (BSA) (Sigma-Aldrich, A7906) in Tris-Buffered Saline Tween-20 (TBST) (Thermo Scientific, AAJ77500K8) for 1 h at room temperature and incubated with primary antibodies overnight at 4 °C (See Supplementary Table 7 for antibody details and RRID numbers). The membrane was then incubated for 1 h at room temperature with horseradish peroxidase-conjugated secondary antibody (Cell Signaling, 7074/7076; Abcam, ab97135) and immune complexes were visualized by enhanced chemiluminescence detection (Millipore, WBLUF0500). See Supplementary Fig. 10 for original blot images.

### RNA Extraction

RNA extraction on human cell lines and mouse organoids was performed using the NucleoSpin RNA Plus extraction kit (Macherey-Nagel, 740984) following the manufacturer’s recommendation. RNA extraction from GEM prostate tumors was performed on frozen samples using the Maxwell 16 LEV simplyRNA Tissue Kit (Promega, AS1280).

### RNA-seq analysis

Specimens were prepared for RNA-seq as described above and RNA quality was verified using Agilent Bioanalyzer 2100 (Agilent Technologies). Paired-end, 50 × 2 cycles sequencing was performed on the HiSeq 4000 instrument. Quality control of raw sequencing reads was performed using FastQC (Babraham Bioinformatics). Low-quality reads were removed using Trimmomatic^33^ with a sliding window size of 4 bp and a quality threshold of 20. The resulting reads were aligned to mm10 using STAR^34^. Reads were sorted and indexed using SAMtools^35^. Transcript abundance was calculated in FPKM using Cufflinks^36^ and in gene counts using HTSeq^37^. Differential gene expression was assessed using DESeq2^38^. For variant calling, GATK’s best practices pipeline^39,40^ was followed including the alignment method as previously described^41^. Reads with less than 30 sequence lengths were removed before alignment. In brief, reads were aligned to the mm10 reference genome with STAR in paired-end and two-pass mode. PCR duplicates were removed using Picard tools and reads were split into exon segments keeping the grouping information by SplitNCigarReads of GATK^39^. Reads were further realigned at known indel positions and the base quality score was recalibrated. Haplotype Caller^39^ was used for calling variants (both single nucleotide variants and indels) from each of the murine tumor tissues. Filtered variants (covered by at least 10× depth) were annotated in Annovar^42^ using RefSeq gene assembly.

### Chromatin immunoprecipitation

N-Myc ChIP-seq was performed as previously described^6^ with modifications. Briefly, primary mouse prostate tumors were harvested and frozen in OCT (Sakura Finetek USA INC, 25608-930). Frozen tumor blocks were sliced into 30 µm thick cryosections. Fifteen sections were collected and crosslinked in 1% methanol-free formaldehyde (Thermo Scientific, 28908) for 10 min at 37 °C. Crosslinking was quenched with 2.5 M glycine for 5 min at room temperature. Fixed samples were washed twice with cold PBS. Pellets were then resuspended in lysis buffer (0.5% SDS, 10 mM EDTA, 50 mM Tris HCl pH 8, protease and phosphatase inhibitors; Thermo Scientific, 78428/78430) and agitated for 20 min at 4 °C. Lysed cells were centrifuged and the supernatant discarded. Nuclei were lysed in a second lysis buffer (10 mM Tris HCl pH 7.5, 150 mM NaCl, 1 mM EDTA pH 8, 1% NP-40, 1% sodium deoxycholate, 0.1% SDS, protease and phosphatase inhibitors) for 15 min on ice. The chromatin was then sheared for 20 min (Diagenode, Bioruptor Pico). Protein-bound sheared chromatin was cleared using magnetic beads (Invitrogen, 10004D) and equal volumes of chromatin were subsequently incubated with 5 µg/sample of IgG control antibody (Santa Cruz Biotechnology, sc-2025) or N-Myc antibody (Santa Cruz Biotechnology, sc-53993) overnight at 4 °C. The immunoprecipitated chromatin was thoroughly washed with different washing buffers (low salt: 150 mM NaCl, 1% Triton X-100, 0.1% SDS, 2 mM EDTA pH 8, 20 mM Tris HCl pH 8, protease inhibitor; high salt: 500 mM NaCl, 1% Triton X-100, 0.1% SDS, 2 mM EDTA pH 8, 20 mM Tris HCl pH 8, protease inhibitor; LiCl: 250 mM LiCl 1% NP-40, 1% sodium deoxycholate, 1 mM EDTA pH 8, 10 mM Tris HCl pH 8, protease inhibitor; and TE: 10 mM Tris HCl pH 8, 1 mM EDTA pH 8) and eluted in 300 µL of fresh elution buffer (100 mM NaHCO3 and 1% SDS). Reverse crosslinking was achieved by adding 45 mM Tris pH 7, 170 mM NaCl, and RNase A to the chromatin and incubating at 65 °C overnight. Chromatin was then incubated with proteinase K and EDTA for 2 h at 45 °C. Following digestion, the DNA fragments were purified using NucleoSpin Gel and PCR Clean-up Kit (Macherey-Nagel, 740609) following the manufacturer’s instructions. DNA libraries were prepared using the Hyper Prep Kit (Kapa Biosystems, KK8502). The libraries were then cleaned using SPRIselect magnetic beads (Beckman Coulter, B23317) and were assessed for purity, quality, and size using DNA High Sensitivity Bioanalyzer chips (Agilent, 5067-4626). Samples that passed quality control were quantified, pooled together, and sequenced. The sequencing was performed by the Weill Cornell Medicine Genomics and Epigenomics Core using an Illumina HiSeq 4000 instrument.

### ChIP-seq analysis

Quality control of raw sequencing reads was performed using FastQC (Babraham Bioinformatics). Low-quality reads were removed using Trimmomatic^33^ with a sliding window size of 4 bp and a quality threshold of 20. The resulting reads were aligned to mm10 using Bowtie2^43^. PCR duplicates introduced during library creation were removed using SAMtools^35^. ChIP-seq peaks were called using MACS2^44^ with default parameters and a *q*-value threshold of 0.0001. Sequencing reads from sonicated input chromatin derived from each individual tumor in each condition were used as a control for peak calling.

### Downstream analysis of sequencing data

Enriched ChIP-seq peak regions were annotated to mm10 genomic features and assessed for the presence of transcription factor motifs +/−100 bp from the ChIP-seq peak using HOMER^45^. Regions of ChIP-seq overlap were defined using BEDTools^46^. ChIP-seq enrichment profile plots and heatmaps were generated using deepTools^47^. Gene set enrichment analysis (GSEA)^48^ was performed using gene sets included in the Molecular Signature Database.

### Single-cell digestion and sequencing

Prostates were dissected from 8-week old mice as previously described^32^, mechanically dissociated to small fragments, and washed. Tissues were digested for 1 h at 37 °C with shaking in 10 mL/gram of a digestion solution comprised of DMEM/F-12 containing 1 mg/mL collagenase (Gibco, 17101-015), 0.2 mg/mL hyaluronidase type VIII (Sigma, H3757), 5 mM calcium chloride, 250 nM A83-01 (Tocris Biosciences, 2939), 10 μM Y-27632 (Sigma, Y0503), 1.25 mM N-acetyl-L-cysteine (Sigma, A7250), 10 mM HEPES (Gibco, 15630-130), 1× GlutaMAX (Gibco, 35050-061), and 1× penicillin/streptomycin (Gibco, 15140-122). Digestion was neutralized with media containing 1× B-27 supplement (Gibco, 17504-044). Additional digestion was performed in TrypLE containing 10 μM Y-27632 while shaking for 15 min at 37 °C. Following neutralization, cells were washed in PBS, filtered through a 70 μm filter, subjected to red blood cell lysis, and filtered through a 35 μm filter before single-cell library preparation. Library preparation was performed at the Genomics Resources Core Facility at Weill Cornell Medicine using the 10× Chromium platform following the manufacturer’s recommended protocols. Briefly, 8000 cells were targeted for 3′ RNA library preparation, multiplexed in a NovaSeq 6000, and sequenced at an average depth of 25,000 reads per cell. Raw sequencing reads were aligned to mm10 reference genome using the *cellranger count* pipeline. Data from individual mice were aggregated using the *cellranger aggr* pipeline and visualized using Loupe Cell Browser. Pseudotime analysis was performed using *Monocle 3*^26,49–52^. RNA velocity analysis was performed using *velocyto*^53^. For single-cell ATAC-seq, 8000 cells were targeted for ATAC library preparation, multiplexed in a NovaSeq 6000, and sequenced at an average depth of 20,000 reads per cell. Raw sequencing reads were aligned to mm10 reference genome using the *cellranger-atac count* pipeline and visualized using Loupe Cell Browser.

### Reduced representation bisulfite sequencing (RRBS)

Methylation profiling was performed on two pairs of adenocarcinomas and poorly differentiated mouse tumors, one of which was from the same mouse with genotype, *Pb-Cre4; Pten*^*f/f*^*; MYCN*^*+/+*^*; Rb1*^*f/f*,^ and the other was from age-matched mice of genotype *Pb-Cre4; Pten*^*f/f*^*; MYCN*^*/+*^*; Rb1*^*f/+*^. DNA was extracted from tissues obtained by coring using 2 mm biopsy punches from histopathologically confirmed foci of OCT frozen tissues. For DNA methylation profiling in cell lines, LNCaP-NMYC cells with RB1 knockdown cells or scrambled shRNA control cells (*n* = 2 replicates) were grown in androgen deprived media (phenol-red free RPMI medium (Gibco, 11835-030) supplemented with 5% charcoal-stripped serum (Gibco, A33821-01) and 1% penicillin/streptomycin (Gibco, 15140-122)) for 96 h and DNA was extracted. Library preparation for RRBS libraries, sequencing, and post-processing of the raw data was performed at the Epigenomics Core at Weill Cornell Medicine^54,55^. The resulting libraries were normalized, pooled, multiplexed, and clustered on single-read flow cells and sequenced for 100 cycles on an Illumina NovaSeq 6000 sequencer. Base call files generated from the sequencer were then demultiplexed and converted to FASTQ files using Illumina *bcl2fastq2* software^56^. Single-end bisulfite-converted reads were aligned using Bismark^57^, following trimming and adapter removal. MethylKit^58^ was used for identifying differentially methylated sites and annotating them to promoters (±1000 bp from TSS), exons, introns, CpG islands, and CpG shores (±2000bp from CpGi). Sites covered by at least 10× coverage were considered for methylation analysis. Sites with at least a 25% difference in methylation between poorly differentiated and adenocarcinomas mice tumors with a 5% False Discovery Rate (FDR) were considered as significantly differentially methylated. A similar analysis was done for LNCaP-NMYC shRB1 and LNCaP-NMYC scrambled control cells.

### Immunohistochemistry (IHC)

Formalin-fixed paraffin-embedded (FFPE) tissue sections were de-paraffinized and endogenous peroxidase was inactivated. Antigen retrieval was accomplished by the Bond Epitope Retrieval Solution 1 (Leica Biosystems, AR9961) at 99–100 °C for 30 min. Following retrieval, the sections were incubated sequentially with the primary antibody for 25 min, post-primary for 15 min, and polymer for 25 min ending with colorimetric development with diaminobenzidine (DAB) for 10 min using the Bond Polymer Refine Detection Kit (Leica Biosystems, DS9800). All histological evaluations and quantifications (including hematoxylin and eosin (H&E)-stained and IHC images) were performed by a board-certified, genitourinary pathologist (B. Robinson) who was blinded to animal genotypes and follow criteria that have previously been described^59^. See Supplementary Table 7 for antibody details and RRID numbers.

### Magnetic resonance imaging (MRI)

Experimental MRI was performed at the Weill Cornell Medicine Citigroup Biomedical Imaging Center on a 7.0 Tesla 70/30 Bruker Biospec small animal MRI system with 450 mT/m gradient amplitude and a 4500 T/m/s slew rate. The animals were anesthetized with isoflurane in oxygen and fixed in the MRI using a nose cone and bite ring. A volume coil was used for transmission and reception. Multi-slice T2 TurboRARE with fat suppression axial and coronal images were acquired using the following parameters: slice thickness = 0.5 mm, the field of view = 40 mm × 40 mm, matrix = 192 × 192, 0.208 mm/pixel × 0.208 mm/pixel, 16 Averages. Coronal: TE –48 ms effective (16 ms with Echo Train Length of 8), TR –2786 ms, but varied if the required number of slices for anatomical coverage changed. Axial: TE –48 ms effective (16 ms with Echo Train Length of 8), TR –4024 ms, but varied if the required number of slices for anatomical coverage changed. The number of slices varied based on the size of the animal. Post-scan analysis was conducted using AnalyzePro software (AnalyzeDirect, Overland Park, KS). Volumetric measurements were obtained by defining a region of interest around the prostate or tumor.

### Reporting summary

Further information on research design is available in the Nature Research Reporting Summary linked to this article.

## Supplementary information

Supplementary Information

Description of Additional Supplementary Files

Dataset 1

Dataset 2

Reporting Summary

## Data Availability

The sequencing data generated in this manuscript have been deposited in the Gene Expression Omnibus (GEO) under accession number GSE151426. Gene sets from the Molecular Signatures Database (MSigDB v7.4) are publicly available [http://www.gsea-msigdb.org/gsea/msigdb/index.jsp]. Source data are available as a Source Data file. Newly generated materials from this manuscript will be shared by the Corresponding Author with Material Transfer Agreements (if applicable). Further information and requests for resources and reagents should be directed to and will be fulfilled by the Corresponding Author, David S. Rickman (dsr2005@med.cornell.edu). Source data are provided with this paper.

## Source data

Source Data
