# Supplementary material for: Temporal evolution of cellular heterogeneity during the progression to advanced AR-negative prostate cancer
Source: Nat Commun. 2021 Jun 7;12:3372. doi: 10.1038/s41467-021-23780-y (PMC8185096; doi:10.1038/s41467-021-23780-y)
Supplement: Supplementary file 1 — Supplementary Information [file 41467_2021_23780_MOESM1_ESM.pdf]

Supplementary Information

Temporal evolution of cellular heterogeneity during the progression to advanced AR-negative prostate cancer

Nicholas J. Brady[1], Alyssa M. Bagadion[1], Richa Singh[1], Vincenza Conteduca[2], Lucie Van Emmenis[1], Elisa Arceci[1], Hubert Pakula[1], Ryan Carelli[1], Francesca Khani[1,3,4,5], Martin Bakht[2], Michael Sigouros[4], Rohan Bareja[6,7], Andrea Sboner[1,4,5,7], Olivier Elemento[4,5,6,7], Scott Tagawa[5,8], David M. Nanus[5,8], Massimo Loda[1,5], Himisha Beltran[2], Brian Robinson[1,3,4,5], and David S. Rickman[1,5,*]

[1] Department of Pathology and Laboratory Medicine, Weill Cornell Medicine, New York, NY, USA
[2] Department of Medical Oncology, Dana Farber Cancer Institute, Boston, MA, USA
[3] Department of Urology, Weill Cornell Medicine, New York, NY, USA
[4] Caryl and Israel Englander Institute for Precision Medicine, New York-Presbyterian Hospital, Weill Cornell Medicine, New York, NY, USA
[5] Meyer Cancer Center, Weill Cornell Medicine, New York, NY, USA
[6] Department of Physiology and Biophysics, Weill Cornell Medicine, New York, NY, USA
[7] Institute for Computational Biomedicine, Weill Cornell Medicine, New York, NY, USA
[8] Department of Medicine, Weill Cornell Medicine, New York, NY, USA
These authors contributed equally: Nicholas J. Brady, Alyssa M. Bagadion

[*] Corresponding Author:
David S. Rickman
Weill Cornell Medicine
413 East 69th St, Room BB1528
New York, NY 10021
+1 (646) 962-3946
dsr2005@med.cornell.edu

SUPPLEMENTARY FIGURE 1

a CRPC + NEPC Patient N-Myc, *RB1*, *PTEN* Status

n=76	*RB1* del. YES	*RB1* del. NO
N-Myc High	19	19
N-Myc Low	20	18

n=76	*PTEN* del. YES	*PTEN* del. NO
N-Myc High	14	23
N-Myc Low	13	26

b

Genotype	Alias
Pb-Cre4$^{+/-}$; *Pten*$^{f/f}$	*P*
Pb-Cre4$^{+/-}$; *Pten*$^{f/f}$; *LSL-MYCN*$^{+/-}$<br>*Pb-Cre4*$^{+/-}$; *Pten*$^{f/f}$; *LSL-MYCN*$^{+/+}$	*PN*
Pb-Cre4$^{+/-}$; *Pten*$^{f/f}$; *Rb1*$^{f/+}$	*PR*$_{het}$
Pb-Cre4$^{+/-}$; *Pten*$^{f/f}$; *Rb1*$^{f/f}$	*PR*
Pb-Cre4$^{+/-}$; *Pten*$^{f/f}$; *LSL-MYCN*$^{+/-}$; *Rb1*$^{f/+}$<br>*Pb-Cre4*$^{+/-}$; *Pten*$^{f/f}$; *LSL-MYCN*$^{+/+}$; *Rb1*$^{f/+}$	*PR*$_{het}$*N*
Pb-Cre4$^{+/-}$; *Pten*$^{f/f}$; *LSL-MYCN*$^{+/-}$; *Rb1*$^{f/f}$<br>*Pb-Cre4*$^{+/-}$; *Pten*$^{f/f}$; *LSL-MYCN*$^{+/+}$; *Rb1*$^{f/f}$	*PRN*

c

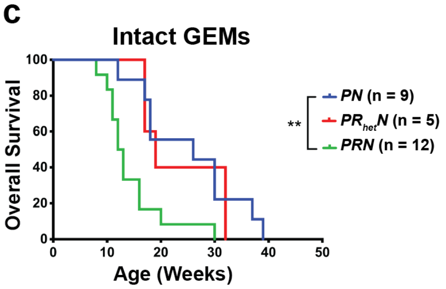

d

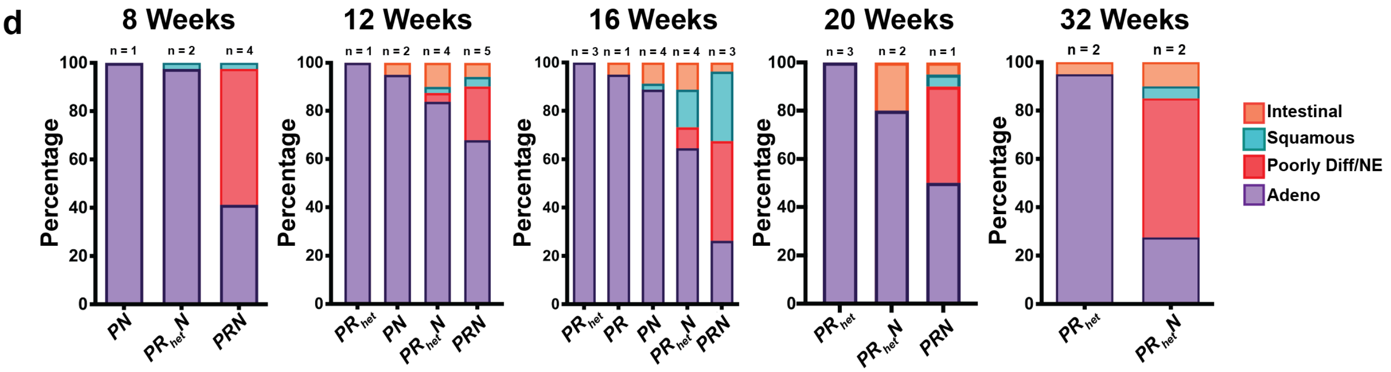

e *PR*$_{het}$*N* (17 weeks)

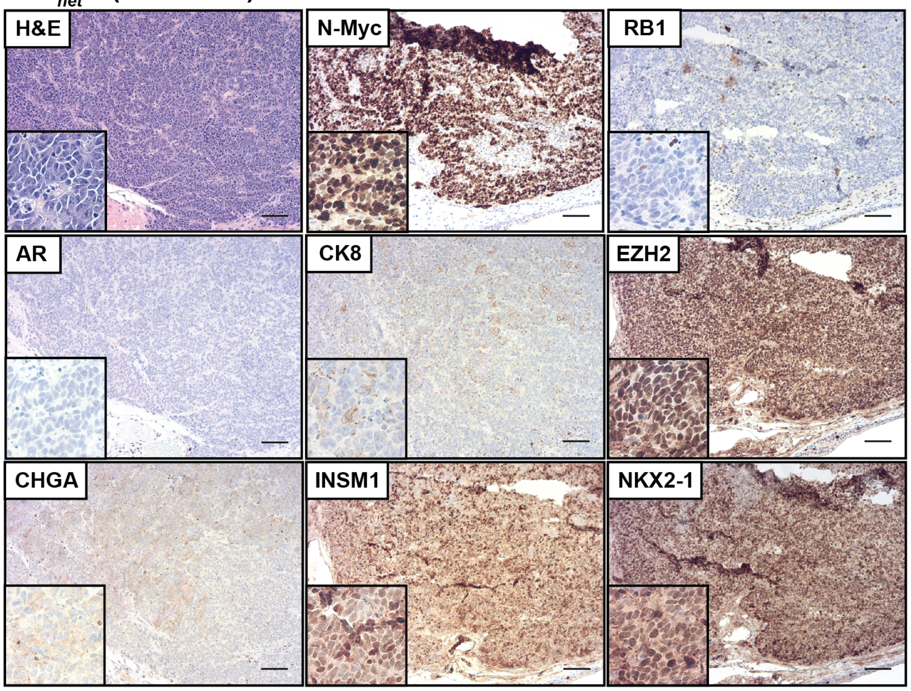

f *PR*$_{het}$*N* (32 weeks)

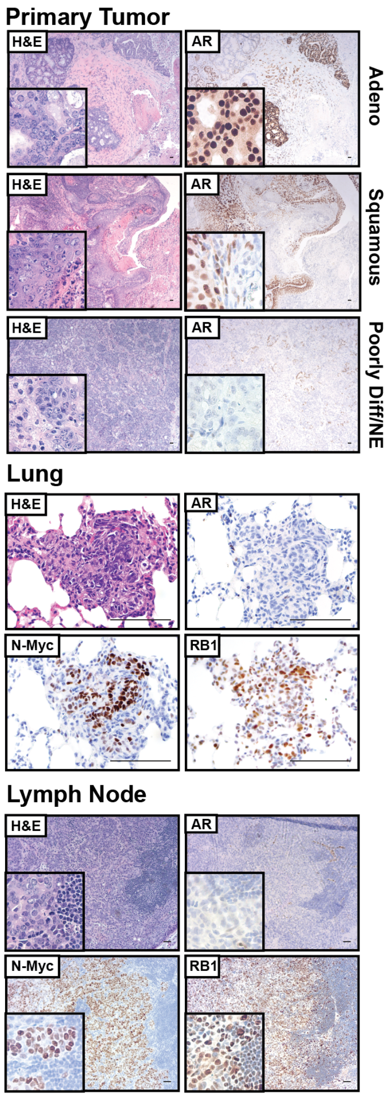

g *PRN* (19 weeks)

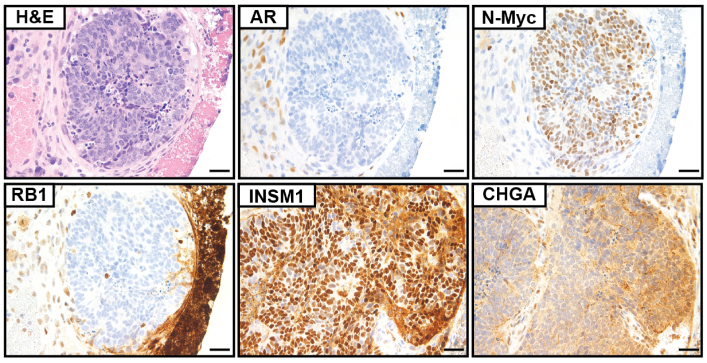

Supplementary Figure 1. N-Myc overexpression and *Rb1* loss drive aggressive prostate cancer. a. Table of patient biopsies grouped according to their tumor histological classification (CRPC or NEPC) and stratified into four categories based on log(FPKM) value of *MYCN* expression (high vs. low) and *RB1* genomic alteration (deletion, yes vs. no) [left] or *PTEN* genomic alteration (deletion, yes vs. no) [right]. **b.** Table of genotype aliases used. **c.** Survival curve of intact GEMs. Survival analysis was performed using the Kaplan-Meier estimator (**p=0.0033, two-sided log-rank test). **d.** Percentage of GEM tumor foci with conventional adenocarcinoma, poorly differentiated/neuroendocrine features, squamous differentiation, and intestinal differentiation based on pathologist assessment. **e.** H&E and IHC staining of NEPC foci for N-Myc, RB1, epithelial markers AR and CK8, reprogramming factor EZH2, and small cell neuroendocrine markers CHGA, NKX2-1, and INSM1. Images are representative of 4 independent mice. Scale bar: 50 µm. **f.** [Top] H&E and IHC of AR on primary tumor showing divergent histology. [Middle/Bottom] H&E and IHC of AR, N-Myc, and RB1 on lung [middle] and lymph node [bottom] metastases. Images are representative of 2 independent mice. Scale bar: 25 µm. **g.** H&E and IHC staining of for N-Myc, RB1, AR, and small cell neuroendocrine markers CHGA and INSM1. Scale bar: 50 µm. Images are representative of 2 independent mice. Source data are provided as a Source Data file.

SUPPLEMENTARY FIGURE 2

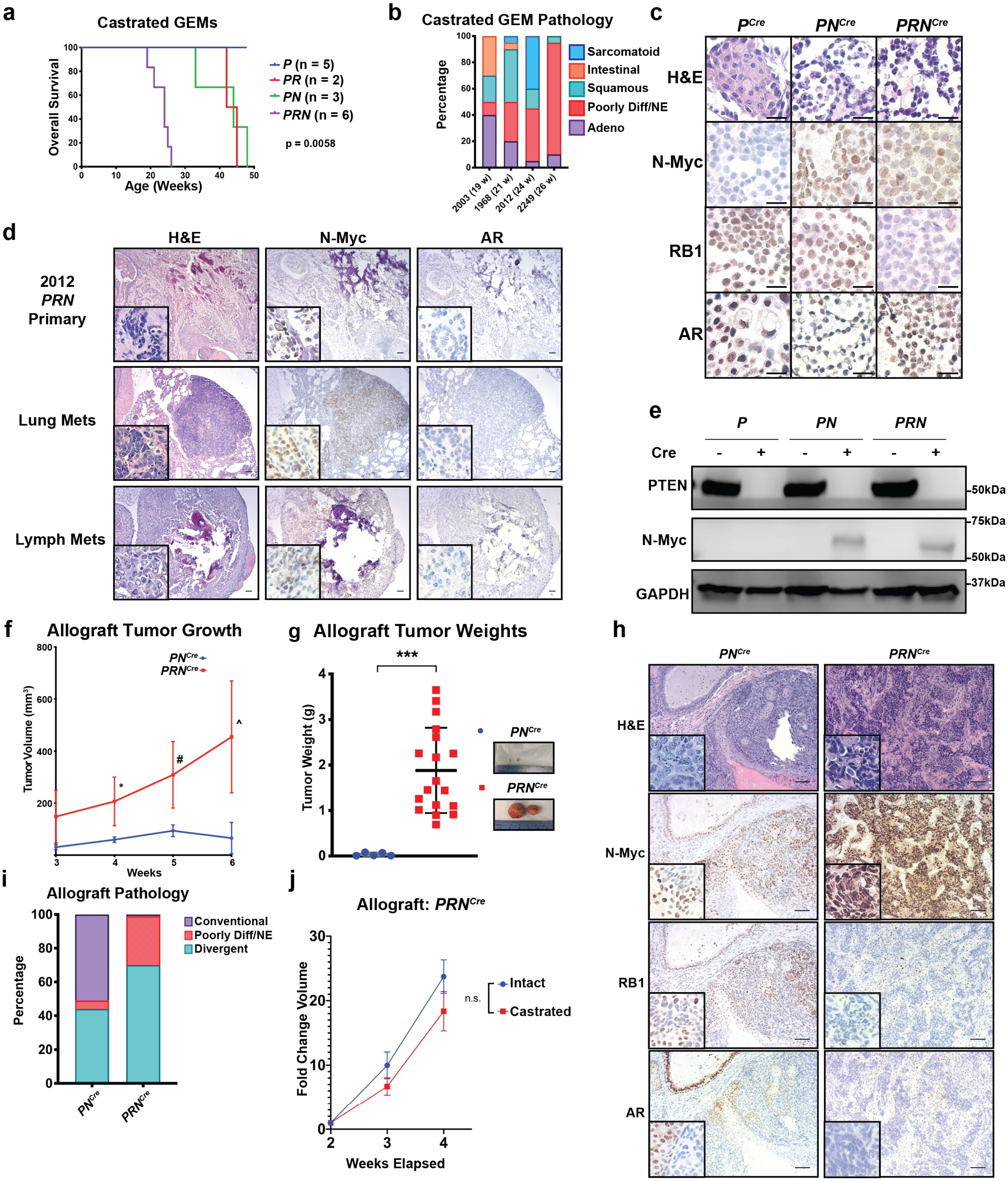

Supplementary Figure 2. *PRN* GEMs are castration resistant. a. Survival curve of castrated GEMs. Survival analysis was performed using the Kaplan-Meier estimator (p=0.0053, two-sided log-rank test). *P* (n=5), *PR* (n=2), *PN* (n=3), *PRN* (n=6). **b.** Percentage of castrated GEM tumor foci with conventional adenocarcinoma, poorly differentiated/neuroendocrine and divergent differentiation (sarcomatoid, intestinal, squamous) based on pathologist assessment. **c.** H&E with N-Myc, RB1, and AR IHC from 3D prostate organoids from the indicated genotypes transduced with Adeno-Cre. Images are representative of organoids derived from 12 independent mice. Scale bar: 25 µm. **d.** Example of H&E, N-Myc and AR IHC from a castrated *PRN* GEM primary tumor and metastases. Images are representative of 6 independent mice. Scale bar: 50 µm. **e.** Immunoblots showing expression of PTEN, N-Myc, and GAPDH to confirm loss of PTEN expression and the induction of N-Myc following Adeno-Cre transduction. Blot is representative of organoids derived from 12 independent mice. **f.** Volume (mm^3) of allografts calculated from caliper measurements from 3 to 6 weeks post-subcutaneous injection. *p=0.0201, $^{\#}$p=0.0161, $^{\wedge}$p=0.0131, two-sided t-test. Data are presented as mean values +/- SD. n=4 independent mice per group. **g.** Allograft tumor weights from PN^{Cre} (n=5) and PRN^{Cre} (n=18) organoids (*** p = 0.0003, two-sided t-test) with images of tumor allografts. Data are presented as mean values +/- SD. **h.** H&E and N-Myc, RB1, and AR IHC from PN^{Cre} and PRN^{Cre} allografts. Left panel includes conventional, squamous, and poorly differentiated tumor foci in PN^{Cre} while the right panel highlights poorly differentiated/NE histology found in the PRN^{Cre} allografts. Representative images from 5 PN^{Cre} and 18 PRN^{Cre} mice. Scale bar: 50 µm. **i.** Tumor histology breakdown in PN^{Cre} and PRN^{Cre} allografts. PRN^{Cre} allografts have divergent differentiation associated with rare, more aggressive cancers such as sarcomatoid and osteoid carcinomas. **j.** Fold change in tumor volume of PRN^{Cre} allografts implanted in intact (n=5) or castrated (n=6) recipient mice. Data are presented as mean values +/- SEM and analyzed using two-sided t-test (p=0.2239). Source data are provided as a Source Data file.

SUPPLEMENTARY FIGURE 3

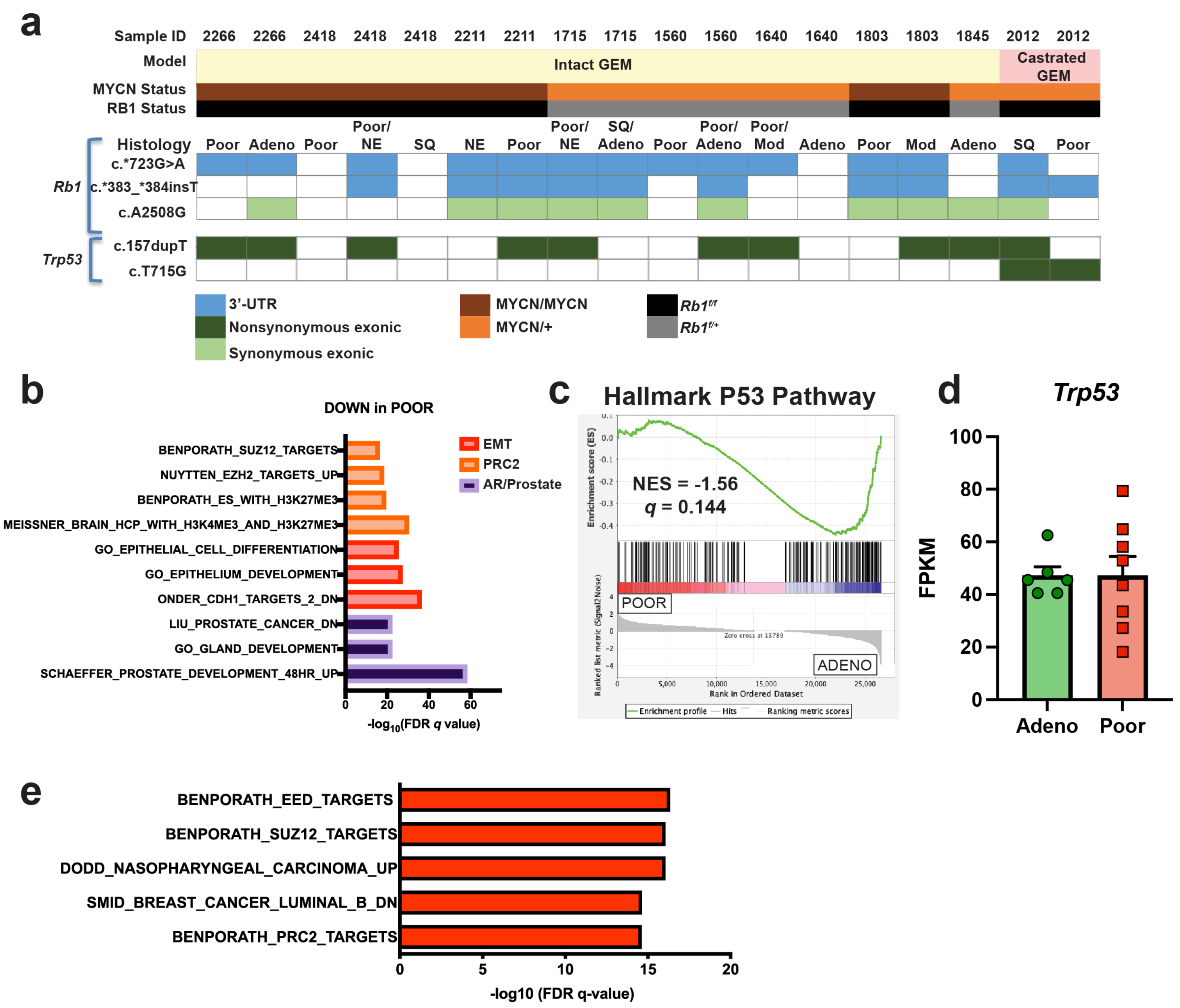

Supplementary Figure 3. Poorly differentiated tumors have molecular signatures similar to clinical NEPC. a. Single nucleotide variants and indels at *Trp53* and *Rb1* in mouse tumors from RNA-seq data. **b.** Top gene sets related to E2F targets, cell cycle, neuronal development, epigenetic reprogramming, epithelial identity, p53 targets, and AR signaling. DOWN: log2FC<-1.5, p<0.05. **c.** GSEA enrichment plot of Hallmark P53 Pathway geneset in poorly differentiated (POOR) vs. adenocarcinoma (ADENO). **d.** Expression of *Trp53* in adenocarcinoma (n=6) and poorly differentiated (n=8) foci. Data are presented as mean values +/- SEM. **e.** GSEA of differentially expressed genes between *Ar+/Cd24a+*/AR Signaling High and *Ezh2+*/AR Signaling Low populations in *PRN* mice in Figure 3f. Source data are provided as a Source Data file.

SUPPLEMENTARY FIGURE 4

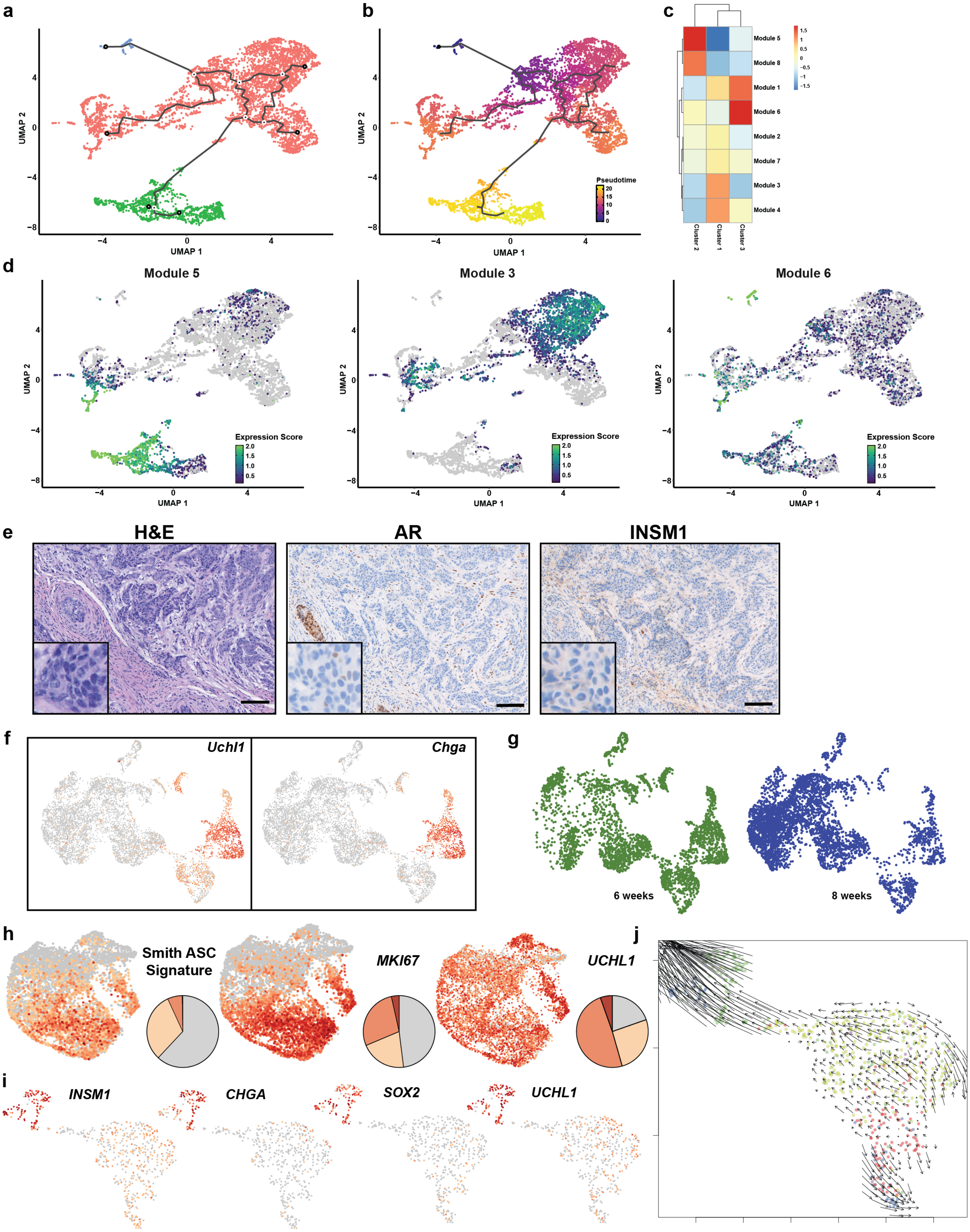

Supplementary Figure 4. Single-cell transcriptomic analysis of *PR* GEM Tumors and NEPC patient sample. a. t-SNE projection of luminal (red), basal (green), and neuroendocrine (blue) populations from *PR* mice (n=3). **b.** Pseudotime trajectories projected onto t-SNE from panel A. **c.** Heatmap and clustering of gene expression modules which vary significantly as a function of pseudotime in *PR* mice. **d.** Expression of genes from modules 5, 3 and 6 projected onto *PR* mice. **e.** H&E and IHC staining of AR and INSM1 in 6-week-old *PRN* mice. Representative images from two independent mice. Scale bar: 100 µm. **f.** Expression of *Uchl1* (left) or *Chga* (right) in luminal and neuroendocrine cells of 6-week-old (n=2) and 8-week-old (n=3) *PRN* mice. **g.** Clustering of luminal and neuroendocrine cells from 6-week-old (n=2) and 8-week-old (n=3) *PRN* mice shown by timepoint (6-week: n=3,521 cells; 8-week: n=4,492 cells). **h.** UMAPs of scRNA-seq data from a metastatic NEPC sample showing Smith ASC signature score, *MKI67,* or *UCHL1* expression. **i.** UMAPs of scRNA-seq data from an NEPC lesion localized in the prostate showing expression of *INSM1*, *CHGA*, *SOX2*, and *UCHL1*. **j.** RNA velocity analysis of scRNA-seq data from panel i. Source data are provided as a Source Data file.

SUPPLEMENTARY FIGURE 5

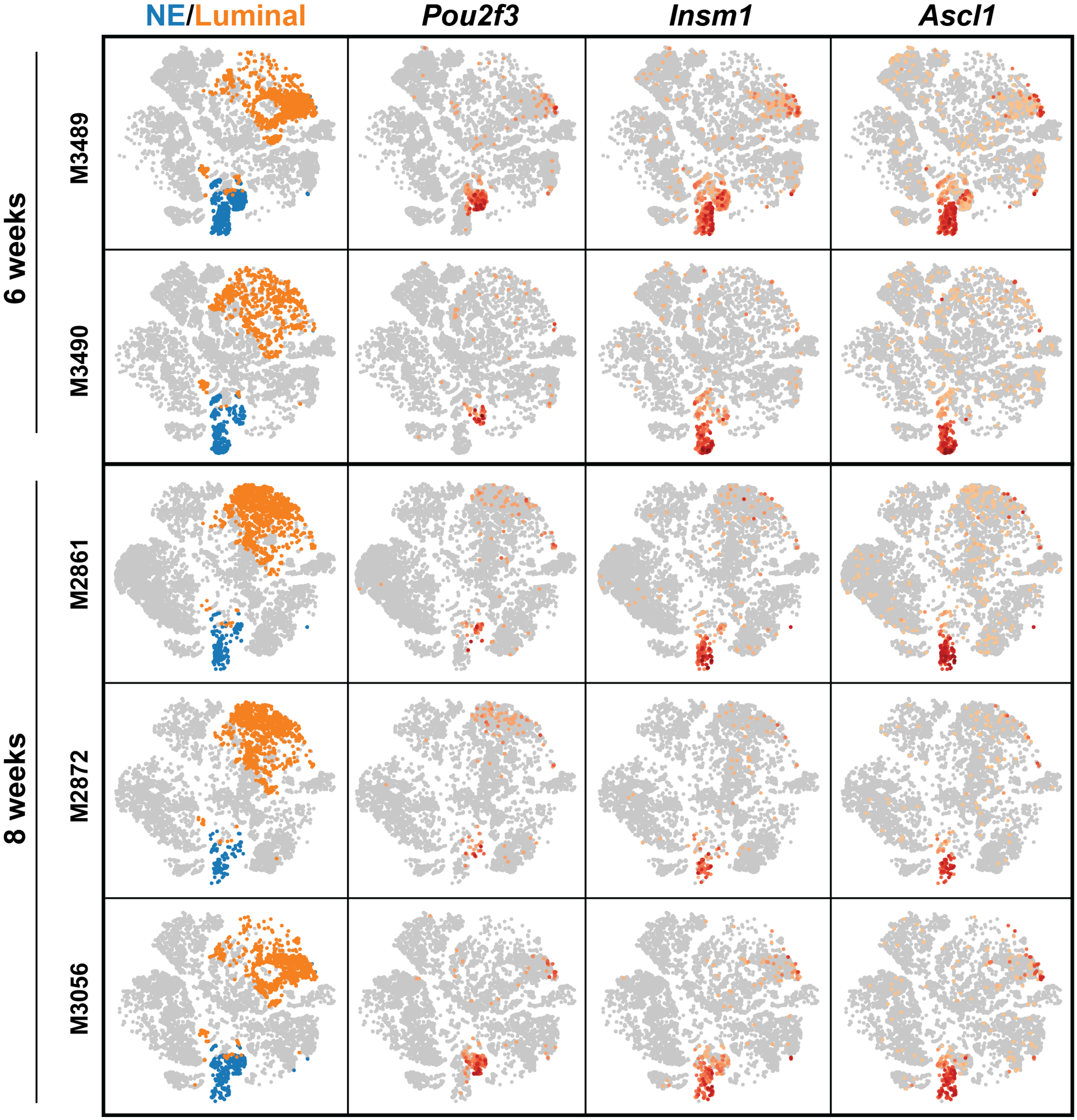

Supplementary Figure 5. scRNA-seq at different time points reveals changing cell populations. t-SNE plots of scRNA-seq data from 6-week (n=2) and 8-week old (n=3) *PRN* mice showing neuroendocrine cells (blue) and luminal epithelial cells (orange) along with expression levels of *Pou2f3*, *Insm1*, and *Ascl1* as indicated.

SUPPLEMENTARY FIGURE 6

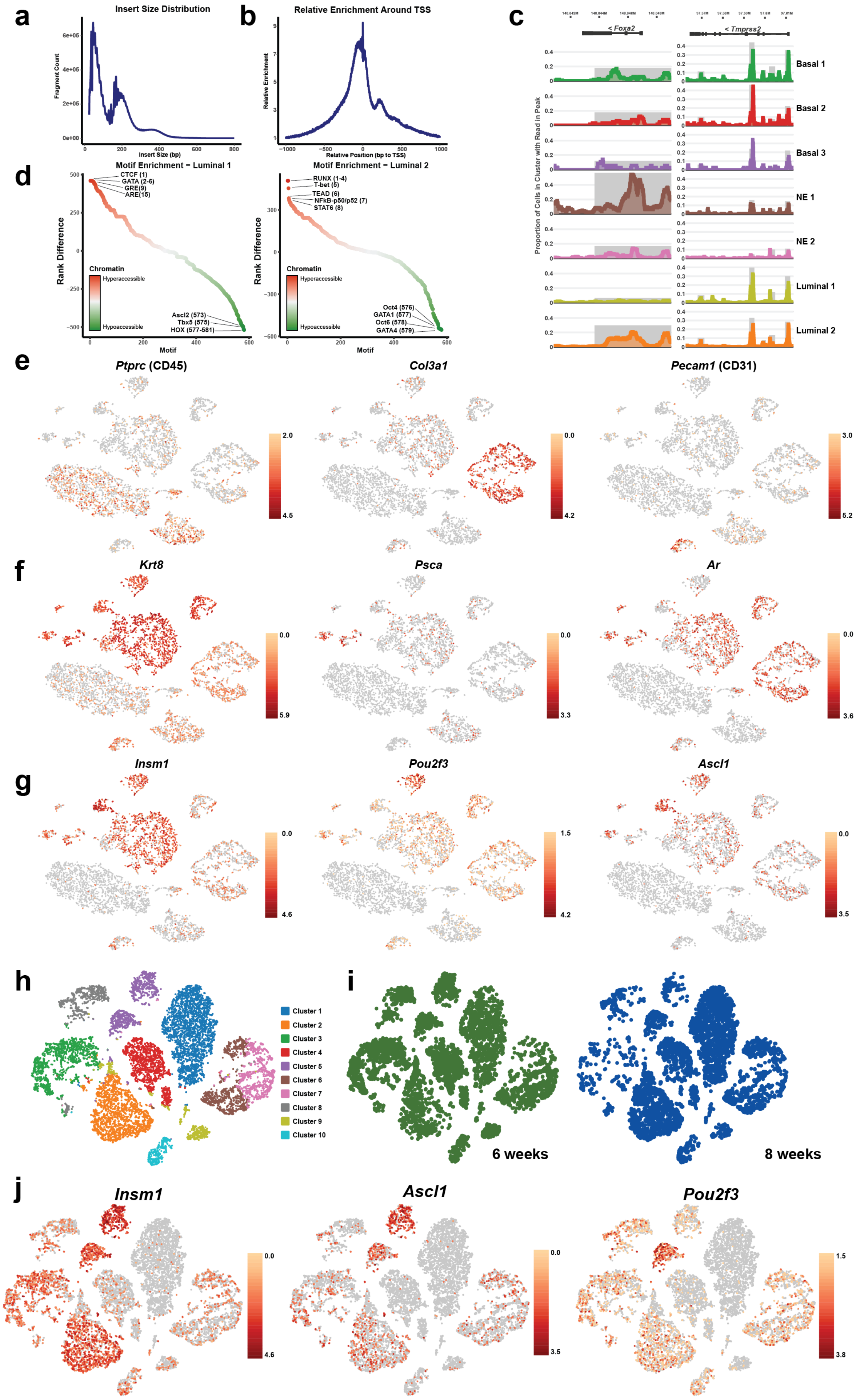

Supplementary Figure 6. Identifying cellular populations by scATAC-seq. a. Insert size distribution histogram from scATAC-seq data revealing expected nucleosome spacing. **b.** Histogram depicting the relative enrichment of accessible chromatin centered at the transcription start site (TSS) of genes. **c.** Chromatin accessibility in the subpopulations at the indicated loci. **d.** Graphs of differential transcription factor motif enrichment in the indicated luminal subpopulations called on regions of hyper- and hypo-accessible chromatin between clusters. **e-g.** Quantification of chromatin accessibility at the indicated loci for markers of **(e)** stromal cells, **(f)** prostate epithelial cells, **(g)** neuroendocrine cells. **h.** Clustering of combined scATAC-seq data from 6-week-old (n=2) and 8-week-old (n=1) *PRN* mice. **i.** Breakdown of clustering data in panel D by timepoint (6-week: n=6,746 cells; 8-week: n=6,059 cells). **j.** Heatmaps of chromatin accessibility at the indicated loci in combined data from 6-week-old (n=2) and 8-week-old (n=1) *PRN* mice. Source data are provided as a Source Data file.

SUPPLEMENTARY FIGURE 7

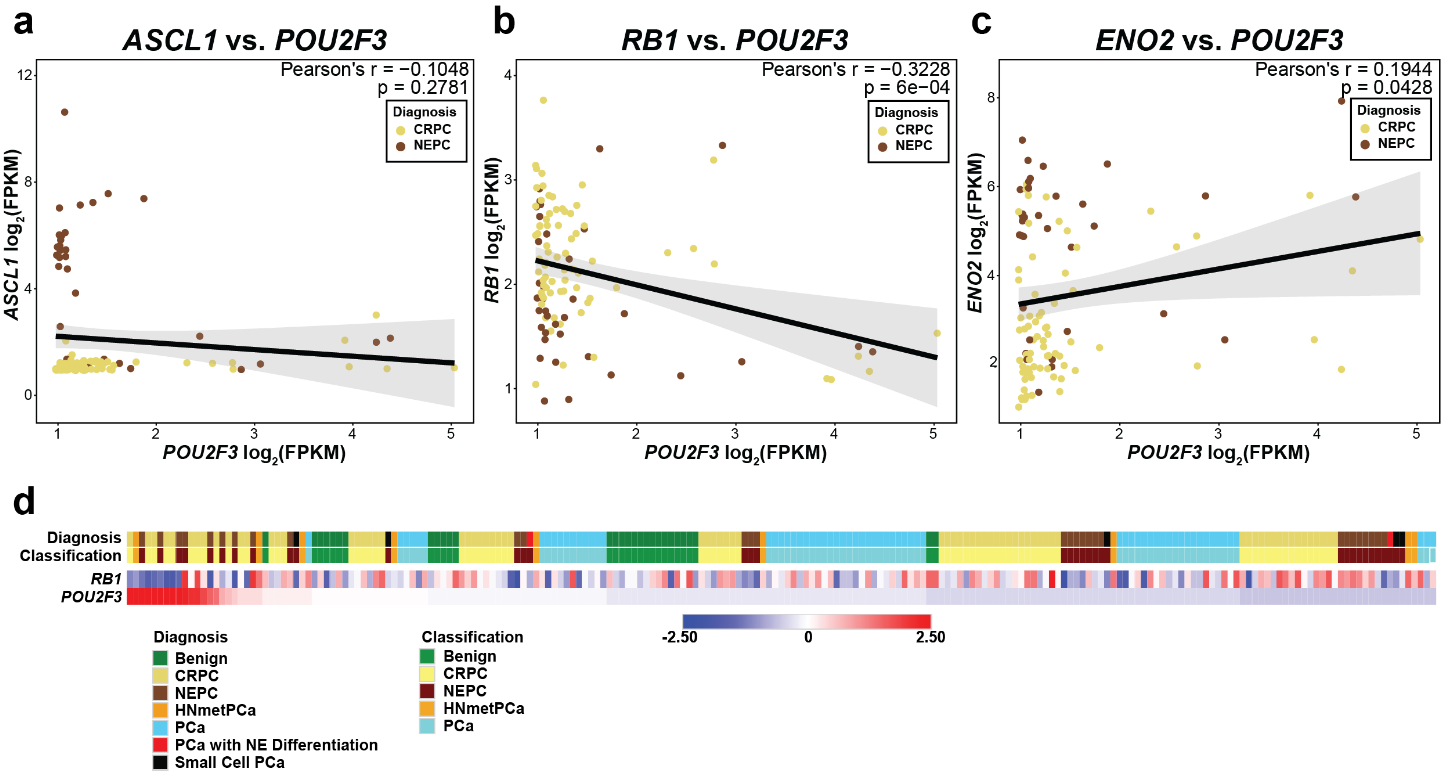

Supplementary Figure 7. Expression of *POU2F3* in clinical samples. a-c. Scatterplot of *ASCL1* (**a**), *RB1* (**b**), and *ENO2* (**c**) versus *POU2F3* expression in clinical samples[2]. Shaded area represents 95% confidence interval. Statistical p values calculated by two-sided Pearson's product-moment correlation. **d.** Heatmap of *RB1* and *POU2F3* expression in clinical samples with diagnosis, stratified by *POU2F3* levels. HNmetPCa = hormone-naïve metastatic prostate cancer. Source data are provided as a Source Data file.

SUPPLEMENTARY FIGURE 8

a

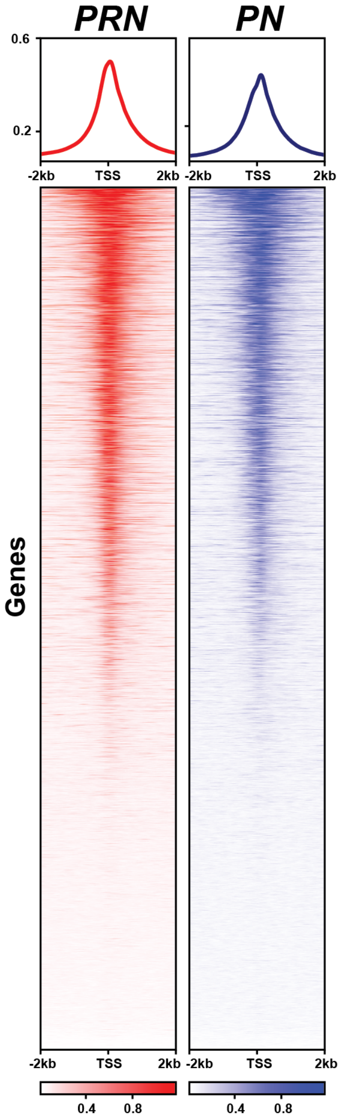

b

***PRN* Specific Sites**

Geneset	FDR(q)
MIKKELSEN_MEF_HCP_WITH_H3K27ME3	$1.15x10^{-180}$
BENPORATH_ES_WITH_H3K27ME3	$1.05x10^{-148}$
BENPORATH_SUZ12_TARGETS	$1.63x10^{-117}$
BENPORATH_EED_TARGETS	$2.24x10^{-111}$
MIKKELSEN_MCV6_HCP_WITH_H3K27ME3	$1.51x10^{-107}$

***PN* Specific Sites**

Geneset	FDR(q)
CHEN_METABOLIC_SYNDROME_NETWORK	$1.85x10^{-23}$
ONDER_CDH1_TARGETS_2_DN	$1.10x10^{-22}$
LEE_BMP2_TARGETS_UP	$1.34x10^{-17}$
NABA_MATRISOME	$4.89x10^{-16}$
LIM_MAMMARY_STEM_CELL_UP	$2.52x10^{-15}$

Supplementary Figure 8. Expansion of the N-Myc cistrome following Rb1 loss. a. Heatmap showing enrichment of N-Myc binding in a 4kb window centered at transcription start sites (TSS) from *PRN* and *PN* tumors. **b.** Top 5 GSEA results from genes uniquely bound by N-Myc in *PRN* and *PN* tumors.

SUPPLEMENTARY FIGURE 9

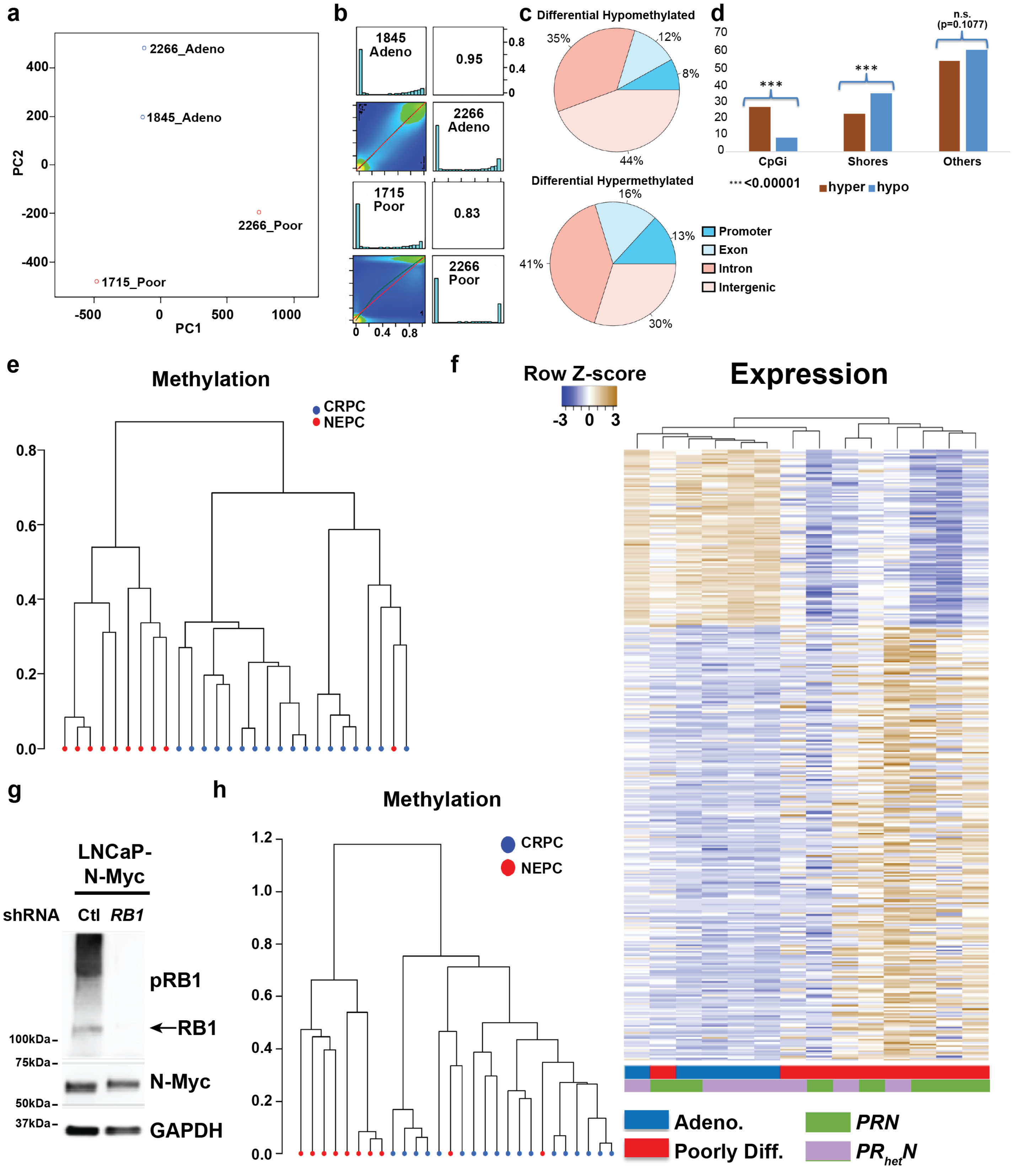

Supplementary Figure 9: Methylation statistics and data from mice tumors. a. PCA analysis with methylation scores of each of the two pairs of poorly differentiated and adenocarcinoma mice tumors. **b.** Correlation density plots showing r^2 values between two adenocarcinomas (top) and two poorly differentiated tumors (bottom). **c.** Distribution of differentially hypomethylated (top) and hypermethylated (bottom) sites (with 5% FDR and 25% difference in methylation in poorly differentiated and adenocarcinoma) across genomic locations. **d.** Distribution of differentially hypomethylated (blue bars) and hypermethylated (brown bars) sites in promoter regions with respect to CpG islands, shores and others. ***$p<0.00001$, chi-squared test. **e.** Cluster map of CRPC (n=18) and NEPC (n=10) samples using methylation scores of all genes (distance method: Correlation, clustering method: Ward D2). **f.** Heatmap with hierarchical clustering of all poorly differentiated tumors and adenocarcinomas from mice based on expression of 360 genes (normalized FPKM values). **g.** Immunoblot showing RB1, N-Myc, and GAPDH expression in LNCaP-N-Myc cells following shCTL or shRB1 treatment. Blot image is representative of 3 independent experiments. **h.** Cluster map of CRPC (n=18) and NEPC (n=10) samples using methylation scores of 242 genes (distance method: Correlation, clustering method: Ward D2). Source data are provided as a Source Data file.

SUPPLEMENTARY FIGURE 10

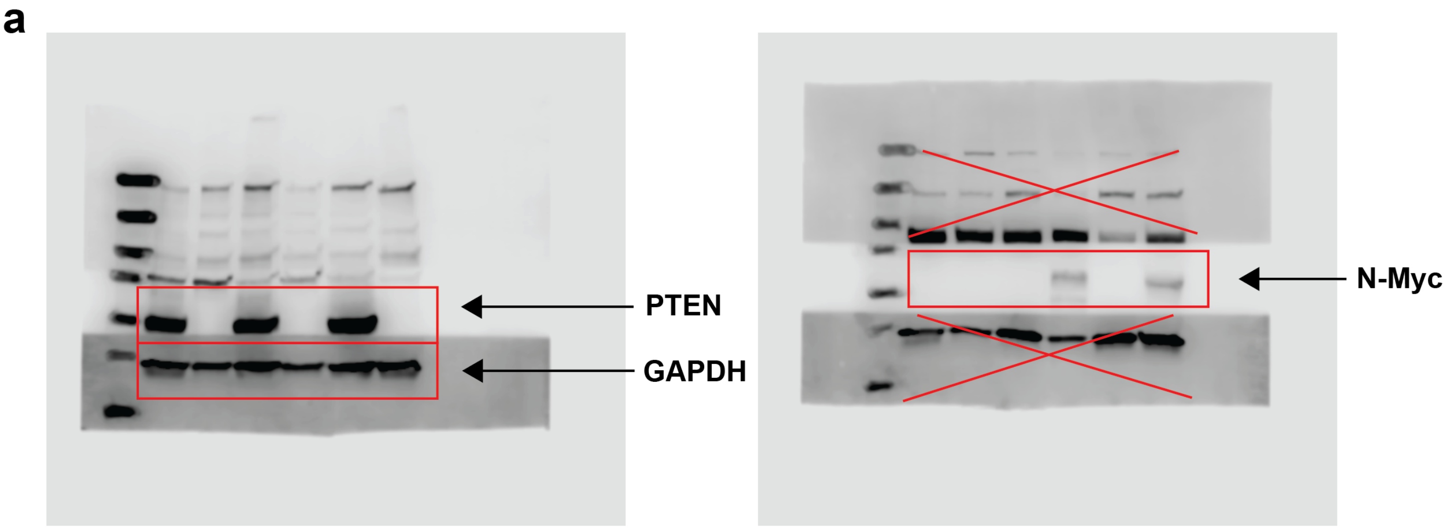

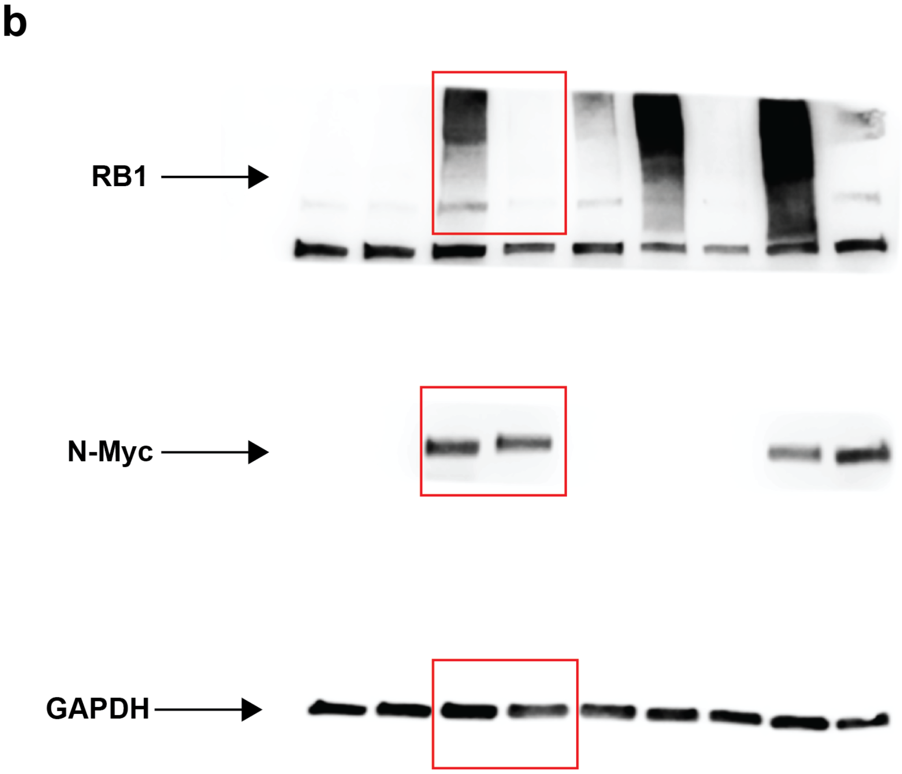

Supplementary Figure 10: Uncropped Western blots. a. Blots from Supplementary Figure 2e. **b.** Blots from Supplementary Figure 9g.

SUPPLEMENTARY TABLE 1

Supplementary Table 1. Summary of Metastatic Lesions Observed in GEMs.

Mouse ID	Intact/Castrated	Gentoype	Alias	Age	Met Descriptions
2346	intact	$Pb\text{-}Cre4^{+/-};Pten^{f/f};LSL\text{-}MYCN^{+/+}$	*PN*	12 weeks	lung, carcinoid tumor
2147	intact	$Pb\text{-}Cre4^{+/-};Pten^{f/f};LSL\text{-}MYCN^{+/-}$	*PN*	33 weeks	lymph node, small focus, poorly differentiated
1826	castrated	$Pb\text{-}Cre4^{+/-};Pten^{f/f};Rb1^{f/f}$	*PR*	41 weeks	pelvic lymph lesions, found dead and could only assess histology of primary tumor which was 100% poorly differentiated<br>liver lesions, found dead and could only assess histology of primary tumor which was 100% poorly differentiated<br>lung lesions, found dead and could only assess histology of primary tumor which was 100% poorly differentiated
1560	intact	$Pb\text{-}Cre4^{+/-};Pten^{f/f};LSL\text{-}MYCN^{+/-};Rb1^{f/+}$	$PR_{het}N$	32 weeks	lung lesions, poorly differentiated
2536	intact	$Pb\text{-}Cre4^{+/-};Pten^{f/f};LSL\text{-}MYCN+/+;Rb1^{f/f}$	*PRN*	8 weeks	lymph node, poorly differentiated<br>lung, 3 micro mets, poorly differentiated
2606	intact	$Pb\text{-}Cre4^{+/-};Pten^{f/f};LSL\text{-}MYCN+/+;Rb1^{f/f}$	*PRN*	9 weeks	lymph node, borderline poorly differentiated and poorly differentiated adjacent areas in transition between tumor types.<br>lung, small lesions, few microscopic foci, about 20 cells each
2266-2	intact	$Pb\text{-}Cre4^{+/-};Pten^{f/f};LSL\text{-}MYCN^{+/+};Rb1^{f/f}$	*PRN*	12 weeks	1 focal lung lesion, poorly differentiated
2130	intact	$Pb\text{-}Cre4^{+/-};Pten^{f/f};LSL\text{-}MYCN^{+/+};Rb1^{f/f}$	*PRN*	12 weeks	lung, 1 micro lesion, poorly differentiated
2229	intact	$Pb\text{-}Cre4^{+/-};Pten^{f/f};LSL\text{-}MYCN^{+/+};Rb1^{f/f}$	*PRN*	12 weeks	lymph node lesions, poorly differentiated
2418-2	intact	$Pb\text{-}Cre4^{+/-};Pten^{f/f};LSL\text{-}MYCN^{+/+};Rb1^{f/f}$	*PRN*	13 weeks	lymph node lesions, poorly differentiated<br>lung lesions, poorly differentiated<br>heart in heart valve lesions, poorly differentiated
1803	intact	$Pb\text{-}Cre4^{+/-};Pten^{f/f};LSL\text{-}MYCN^{+/+};Rb1^{f/f}$	*PRN*	16 weeks	lung lesions, poorly differentiated
2312	intact	$Pb\text{-}Cre4^{+/-};Pten^{f/f};LSL\text{-}MYCN^{+/-};Rb1^{f/f}$	*PRN*	21 weeks	lymph node poorly diff, full of mets<br>liver lesions, poorly differentiated<br>lung lesions, poorly differentiated, a few large
2249	castrated	$Pb\text{-}Cre4^{+/-};Pten^{f/f};LSL\text{-}MYCN^{+/-};Rb1^{f/f}$	*PRN*	26 weeks	lymph node lesions, poorly differentiated<br>liver lesions, poorly differentiated<br>lung lesions, poorly differentiated<br>kidney soft tissue mets in kidney w/in capsule
2012	castrated	$Pb\text{-}Cre4^{+/-};Pten^{f/f};LSL\text{-}MYCN^{+/-};Rb1^{f/f}$	*PRN*	24 weeks	lung lesions in every lobe; poorly differentiation which was not found in primary tumor but may have been missed; some bony sarcomatoid differentiation<br>intestine lesion in mesentary; bony sarcomatoid differentation; carcinoid neuro differentation<br>liver lesion- some focal sarcomatoid differentation on surface<br>bony sarcomatoid differentation on surface of kidney
1975	castrated	$Pb\text{-}Cre4^{+/-};Pten^{f/f};LSL\text{-}MYCN^{+/-};Rb1^{f/f}$	*PRN*	24 weeks	lung lesions, poorly differentiated<br>8 liver lesions, poorly differentiated
2211	intact	$Pb\text{-}Cre4^{+/-};Pten^{f/+};LSL\text{-}MYCN^{+/+};Rb1^{f/f}$	No alias	19 weeks	lymph lesions, large & small cell NEPC and some poorly differentiated<br>liver lesions, large & small cell NEPC and some poorly differentiated<br>lung lesions, large & small cell NEPC and some poorly differentiated<br>kidney surface lesion, large & small cell NEPC and some poorly differentiated
2191	intact	$Pb\text{-}Cre4^{+/-};Pten^{f/+};LSL\text{-}MYCN^{+/+};Rb1^{f/f}$	No alias	36 weeks	lymph lesions, poorly differentiated

SUPPLEMENTARY TABLE 2

Supplementary Table 2. Top 50 Marker Genes from Each Cluster in Figure 3b.

Cluster 1	Cluster 2	Cluster 3	Cluster 4	Cluster 5	Cluster 6	Cluster 7	Cluster 8	Cluster 9	Cluster 10
G0s2	Cym	1500015O10Rik	Svs3b	Fcgr2b	Akr1b7	Gm26917	Tm4sf20	Sox17	Ascl1
Mmp9	Ffar4	Col3a1	Svs3a	Cybb	Spink8	Atg9b	Tgm3	Selp	Sox1
S100a8	Serpinb11	Col5a2	Svs6	Fcrls	Serpina1f	AL732506.1	Car1	Emcn	Nefl
S100a9	Krt6a	Col6a3	Sva	Fcgr1	Crisp1	Ccnb1ip1	Clca4b	Adgrl4	Syt1
Csf3r	Sult1c2	Prrx1	Svs1	C1qb	Crygc	mt-Nd6	Sult1b1	Myct1	Stmn3
Acod1	Wincr1	Dpt	Ceacam10	C1qc	Defb30	Gm15675	Sult1d1	Plvap	Insm1
Ccl3	Gabrp	Ddr2	A630095E13Rik	C1qa	Defb43	Gm48708	Muc3	Cdh5	Rtn1
AC110211.1	Tm4sf4	Col5a1	Spinkl	C3ar1	Aqp2	Rgs11	Clec2h	Gpihbp1	Crmp1
Stfa2l1	Gjb4	Tnfaip6	9530003J23Rik	Nkg7	Acbd7	AC153364.1	Ceacam20	Cldn5	Elavl4
Gm5483	Arl14	Serping1	Svs5	Lyz2	Sptssb	B430010I23Rik	Sycn	Cyyr1	Elavl3
Retnlg	Wfdc18	Mdk	Hba-a1	Gpr183	Cited1	Gm47283	Saa1	Ptprb	Ina
Trem1	R3hdml	Fbn1	Svs4	Cd3g	Vsig2	Egfros	Aqp8	Tmem252	Scn9a
Il1f9	Rgs4	Cpxm1	Hbb-bs	Ccr2	Ly6f	Gm43221	Dmbt1	Grrp1	Foxa2
Clec4d	Vtcn1	Myl9	Hba-a2	Ccr5	Defb42	Lars2	Cdhr5	Fam167b	Ovol3
Hdc	Sftpd	Ptgis	Svs2	Ccr7	Gstm1	mt-Atp6	Mgat4c	Egfl7	St18
E230032D23Rik	Cxcl15	Bgn	Dnase2b	Ly86	Gm37223	Lama5	Ces2c	Tie1	Rab3c
Clec4e	Krt4	Postn	Hbb-bt	Cd83	Eddm3b	Coro6	1810065E05Rik	Esam	Chga
Cxcr2	Fabp1	Ptx3	Igkc	Pld4	Pantr1	Gm19557	Car4	Robo4	Celf3
Ccrl2	Cxcl5	Sfrp2	Pate4	Aif1	Wfdc10	Fer1l4	Slc26a3	Flt1	Pcsk1
Slfn1	Atoh1	Ctsk	Jchain	Trem2	Fmo5	mt-Co3	Ly6g	Pecam1	Scn3a
Il1r2	Kctd14	Penk	Gm17689	Ms4a7	Wfdc15b	Als2cl	Cyp2d34	Aqp1	Hcn1
Ccl4	Slc39a4	Svep1	Igha	Ms4a4c	9530053A07Rik	mt-Nd2	2310079G19Rik	Aplnr	Tmem163
Ptgs2os2	Urah	Mfap2	Cdo1	Ms4a6c	Kcnj16	Chsy3	Prss30	Kdr	Scg3
Wfdc21	Klk1b11	Mmp23	Nefh	Ms4a6b	Akr1cl	mt-Co2	Ms4a8a	Icam2	Bhlhe22
Fpr2	Krt78	Mxra8	Orm3	Cd3d	Sbpl	mt-Atp8	2010109I03Rik	Mmrn2	Srrm3
Ccr1	Msln	Cpz	Gm5420	Cd48	Irx1	mt-Nd4l	Cdhr2	Adgrf5	Slc35d3
Nlrp3	Prr9	Htra3	Olfr1033	Msr1	Dnmt3l	D830024N08Rik	Slc51b	Ackr1	Kcnk16
Hcar2	Nlrp10	Pdgfra	Elf5	Runx3	Pbsn	mt-Nd4	Krt20	Sox7	Snca
Asprv1	Ppp1r1b	Pcolce	Iglv1	Ms4a4b	Sbp	Hist1h1d	Lypd8	Apold1	Soga3
Slc7a11	Ttr	Medag	Gpr150	H2-Eb1	2310057J18Rik	mt-Cytb	Mep1b	S1pr1	Gnat3
Slfn4	Lpcat4	Col1a2	Cyp4a12a	Il7r	Prol1	Gm29237	Tnni1	Ccl21a	Elavl2
4833407H14Rik	Reg1	Wnt2	Tnfrsf17	Csf1r	Odam	Sytl2	Tmem236	Pcdh17	Gng13
Cyp4f18	Reg3g	Rarres2	Mzb1	Il21r	Glb1l3	Zfp692	Clca4a	Mmrn1	Htr3a
Clec5a	Gpx2	Mfap5	Iglv3	Mgl2	Shh	Grk3	Hao2	Adcy4	Snap25
Fpr1	Cbr2	C1s1	2010007H06Rik	Napsa	Abcg2	4631405K08Rik	Mep1a	Ushbp1	Pex5l
Ptafr	Klk1b24	Mgp	Ccr10	H2-DMb2	Snx31	Ttc6	Ces2a	Rasgrp3	Pclo
Trem3	Btc	Fxyd1	Lmo3	Cd86	Ren1	Malat1	Cyp2d10	Cd93	Avil
Mirt1	Onecut2	Rcn3	Lpo	Pf4	Pip	Gm4876	Gm5485	Rasip1	Uchl1
Cd300lf	Aspa	Serpinh1	Bpgm	Cd72	Smpx	mt-Nd3	Glod5	Nos3	Kcnc1
Samsn1	Psapl1	Spon1	Cacna1s	Il2rb	Tgm4	Gm26518	Trpm6	Eng	Adcy1
Trim30b	Cxcl17	Cpxm2	Cyp2j13	Cfp	AU021092	E530011L22Rik	Zg16	Ramp2	Nrxn1
Ankrd33b	Barx2	Tcf21	Ggt1	Klrd1	C1rb	mt-Nd5	Ifi27l2b	Unc45b	Clstn3
Tlr6	Krt16	Bicc1	Osbp2	H2-Oa	9530002B09Rik	Hist1h4f	Slc39a5	Klhl4	Iapp
Cd300ld	Slc10a2	Col6a2	Tulp2	P2ry10	Aldh3a1	4933423P22Rik	Myo1a	She	Nefm
Gm20406	Aldh1a3	Col6a1	Kcna2	Ptprcap	Upk1a	Upk2	Cyp4f14	Arhgef15	Bmx
Plek	Rad51b	Dcn	Upb1	Cd7	Gsta2	4930503B20Rik	Slc15a1	Lyve1	Rgs13
Rdh12	Rnf183	Lum	Shisa4	Cd3e	Apoc1	Col25a1	Eno3	Bcl6b	Prox1os
Siglecf	Gp2	Sfrp1	Anxa9	Clec4a3	Upk2	Paxbp1	Hsd17b2	Cd300lg	Fam19a4
F630028O10Rik	Vsig1	Pdgfrl	Apol6	Cd79a	Smr2	AC149090.1	Ptprh	Esm1	Cnpy1
Themis2	Slc12a2	Mmp2	Doxl2	Sh2d2a	Ldhb	Gm26699	Muc13	Sox18	Cplx2

SUPPLEMENTARY TABLE 3

Supplementary Table 3. Top 50 Differentially Expressed Genes Identified in Intermediate Population

Gene Name	log2(FC)	p-value*
Ovol3	7.9670	9.81E-167
Bmx	6.9732	5.07E-126
Lrmp	6.8059	2.10E-121
Plcb2	7.2779	5.70E-118
Fam19a4	7.1240	1.71E-117
1810046K07Rik	7.2792	1.71E-117
Gnat3	7.8565	1.06E-113
Gng13	6.8497	1.32E-113
Rgs13	6.7108	2.04E-105
Pcdh20	7.4031	2.26E-105
Avil	6.1105	3.04E-102
Tmem132e	7.0481	3.92E-92
Trpm5	6.6455	6.18E-92
Pou2f3	5.9098	3.04E-91
Ascl2	6.9124	6.36E-91
Lipc	6.3453	2.87E-88
Gnb3	7.3230	9.19E-86
C2cd4a	5.9939	9.19E-86
Matk	5.9547	2.33E-84
Rgs8	5.6319	1.67E-77
Utf1	5.3374	1.14E-76
Pvalb	9.2012	2.35E-75
Nxph4	6.5766	7.81E-75
Fam159b	5.8654	1.14E-73
Sv2c	5.4337	7.27E-72
Rcan2	6.0423	5.44E-71
Kcnq3	6.0638	3.38E-70
Mctp1	5.3484	7.45E-67
Ankrd63	6.4883	1.64E-63
Lrrc6	7.2842	4.14E-62
Il10	5.0666	5.42E-57
Alox5	5.5848	1.76E-55
Vav1	4.5407	7.36E-54
Ly6g6f	6.1244	2.24E-53
Zmat4	5.7869	1.28E-52
Ltc4s	4.4019	1.87E-51
Apela	5.7868	1.95E-51
Gm26829	6.8121	5.38E-51
Chn2	4.1698	1.36E-50
Cdkl1	5.1299	1.52E-50
Hpgds	4.2677	3.61E-50
Myt1l	5.0144	2.17E-49
Alox5ap	4.3235	2.52E-49
Colca2	4.3190	2.24E-48
Hoxaas3	4.4456	3.56E-48
1500009L16Rik	4.2451	8.77E-48
Stx1b	5.5353	9.96E-48
Ackr4	5.4588	1.41E-46
Spib	5.1068	1.83E-46
Lrrtm1	6.3013	3.60E-46

* Benjamini-Hochberg adjusted two-sided t-test

SUPPLEMENTARY TABLE 4

Supplementary Table 4. Mutations and Deletions from Clinical NEPC Sample in Figure 4h

Site	Gene	Locus	Type	Coding Change	Clonal Frequency
Prostate	*PTEN*	chr10:89,536,129-90,122,394	Focal Deletion	---	---
Prostate	*TP53*	chr17:7,577,121	Missense	p.R273C	76.32%
Liver	*PTEN*	chr10:89,536,129-90,122,394	Focal Deletion	---	---
Liver	*TP53*	chr17:7,577,121	Missense	p.R114C	95.24%
Liver	*RB1*	chr13:49,027,168	Nonsense	p.R579X	85.71%

SUPPLEMENTARY TABLE 5

Supplementary Table 5. Top 50 Differentially Accessible Genes between NE1 and NE2 in Figure 5a.

NE1 Cluster

Gene Name	log2(FC)	p-value*
Tns3	1.7466	4.84E-17
Pkd1l1	2.6116	1.11E-15
Foxa2	1.7261	2.40E-14
Efcab1	2.5215	1.26E-13
Spink13	2.1931	1.03E-12
Scn3a	2.1894	5.24E-12
Mical2	1.5092	5.24E-12
Adcy1	1.7915	2.03E-11
Vwce	2.1557	2.42E-11
Ednra	2.6242	8.47E-11
Ghr	1.9130	8.47E-11
Neto1	1.8626	1.95E-10
Cacna2d1	1.5473	3.69E-10
Pde8b	1.8804	3.69E-10
Kcnb2	1.8582	3.72E-10
Cbr4	1.4721	7.31E-10
Rimbp2	2.1445	1.79E-09
Palmd	1.6354	5.10E-09
Homer2	1.5265	2.10E-08
Drd5	1.6289	2.45E-08
Lrp1b	2.2846	3.19E-08
Tll1	2.0761	4.74E-08
Tmem163	1.5022	6.06E-08
Astn2	1.7087	6.16E-08
Eps8	1.3625	7.29E-08
Adh7	1.7247	7.61E-08
Thsd7a	1.5994	1.07E-07
Tnp1	1.5295	1.08E-07
Slc35d3	1.3917	1.85E-07
Gad1	1.4383	2.08E-07
Sox5	1.3674	2.13E-07
Clstn2	1.3382	2.13E-07
RP23-168H19.1	1.3748	2.65E-07
Col8a1	1.5404	2.76E-07
Pabpc2	1.4261	2.85E-07
Obsl1	1.5952	3.40E-07
Khdrbs3	1.5116	3.42E-07
Enox1	1.4190	4.10E-07
Negr1	1.8891	4.50E-07
Fam171a1	1.3325	4.60E-07
Med10	1.8123	5.67E-07
Ror2	1.1537	5.99E-07
Dusp16	1.2449	6.09E-07
Nrp1	1.2576	6.34E-07
Arhgap24	1.1700	7.49E-07
Prkd1	1.4528	8.26E-07
Sema3e	1.3541	9.79E-07
Gm9767	1.9274	1.06E-06
Kcne4	1.8487	1.11E-06
Stoml3	1.4707	1.20E-06

* Benjamini-Hochberg adjusted two-sided t-test

NE2 Cluster

Gene Name	log2(FC)	p-value*
Pou2f3	2.5828	4.87E-22
Sel1l2	4.5081	1.86E-19
Olfr1414	2.5229	1.36E-13
Spib	2.0517	6.48E-12
Epha6	2.1172	2.53E-11
Csmd2	2.2128	9.57E-11
Vav1	1.5731	1.11E-10
Plcb2	1.6650	5.47E-10
Ascl2	1.8763	5.47E-10
Hck	1.7090	8.56E-10
Hpgds	1.9935	5.20E-09
Fam19a4	1.5195	5.20E-09
RP23-193D1.3	1.9010	6.94E-09
Tas2r134	1.8208	1.00E-08
Ccdc129	1.7154	1.66E-08
Gsc	1.6453	1.69E-08
Trhde	1.3554	1.71E-08
Adora1	1.5484	2.05E-08
Cpvl	1.7649	2.32E-08
Sox9	1.2575	9.83E-08
Aox4	2.0572	1.12E-07
Gng13	1.6987	1.33E-07
Mctp1	1.2850	1.61E-07
Mcub	1.4872	2.82E-07
Gm10577	1.4931	2.84E-07
Ly6g6e	2.2017	3.15E-07
Mgll	1.2047	4.43E-07
Apobec1	1.6483	5.63E-07
Stk38	1.3059	8.26E-07
Gucy2d	1.5791	8.28E-07
Vstm2l	1.4209	8.77E-07
Cd2	1.5039	1.11E-06
Gnb3	1.5702	1.13E-06
Csf2	1.7135	1.19E-06
Tmem132e	1.3728	1.22E-06
Nod2	1.4667	2.00E-06
Pamr1	1.4686	2.20E-06
Sash1	1.1039	2.23E-06
Spdef	1.1099	2.48E-06
Lrmp	1.2729	2.57E-06
Nek11	1.3345	2.77E-06
Rassf6	1.1721	3.44E-06
Gfi1b	1.4731	3.47E-06
Gsg1l	1.1474	3.49E-06
Fes	1.2504	5.33E-06
RP23-421B5.3	1.1709	5.84E-06
Fam19a1	1.3128	6.23E-06
Actr8	1.4293	7.42E-06
Ppargc1b	1.0715	7.45E-06
Alox5	1.4831	7.80E-06

* Benjamini-Hochberg adjusted two-sided t-test

SUPPLEMENTARY TABLE 6

Supplementary Table 6. Organoid Media Components and Recipe

Factor	Vendor/Catalog #	Final Concentration
B27 Supplement	Gibco, 17504-044	1x
N-acetyl-cysteine	Sigma, A7250	1.25 mM
Recombinant mouse EGF	Gibco, PMG8043	50 ng/mL
Noggin-containing CM	conditioned media	10%
R-spondin-containing CM	conditioned media	5%
A83-01	Tocris, 2939	500 nM
DHT	Sigma, D-073	1 nM
Y-27632	Sigma, Y0503	10 mM
Primocin	Invivogen, ant-pm-1	100 mg/mL
GlutaMAX	Gibco, 35050-061	1x
HEPES	Gibco, 15630-130	10 mM
Pen/Strep	Gibco, 15140-122	1%
Advanced DMEM/F-12	Gibco, 12634-010	500 mL

SUPPLEMENTARY TABLE 7

Supplementary Table 7. Antibody Information

Antibody	Vendor/Catalog #	RRID #	Dilution
Mouse anti-N-Myc (WB/ChIP-seq)	Santa Cruz: sc-53993	AB_831602	1:200 (WB); 5 μg (ChIP)
Rabbit anti-N-Myc (IHC)	Cell Signaling: 51705	AB_2799400	1:100 (IHC)
Mouse anti-EZH2	BD Bioscience: 612667	AB_399911	1:50 (IHC)
Chicken anti-GAPDH	Millipore: AB2302	AB_302962	1:10,000 (WB)
Rabbit anti-RB1	Abcam: ab181616	AB_2848193	1:500 (IHC); 1:1,000 (WB)
Rabbit anti-AR	Abcam: ab108341	AB_10865716	1:100 (IHC)
Mouse anti-INSM1	Santa Cruz: sc-377428	AB_2848191	1:100 (IHC)
Rat anti-Keratin 8	DSHB: TROMA-I	AB_531826	1:400 (IHC)
Rabbit anti-CHGA	Immunostar: 20085	AB_572227	1:1,000 (IHC)
Mouse anti-NKX2.1	ThermoFisher: MS-699	AB_142011	1:200 (IHC)
Rabbit anti-PTEN	Cell Signaling: 9188	AB_2253290	1:1,000 (WB)
Mouse anti-POU2F3	Santa Cruz: sc-293402	AB_2890011	1:100 (IHC)
Anti-mouse IgG	Santa Cruz: sc-2025	AB_737182	5 μg (ChIP)
Anti-mouse IgG-HRP	Cell Signaling: 7076	AB_330924	1:5,000 (WB)
Anti-rabbit IgG-HRP	Cell Signaling: 7074	AB_2099233	1:10,000 (WB)
Anti-chicken IgY-HRP	Abcam: ab97135	AB_10680105	1:10,000 (WB)
