# Supplementary material for: Temporal evolution of cellular heterogeneity during the progression to advanced AR-negative prostate cancer
Source: Nat Commun. 2021 Jun 7;12:3372. doi: 10.1038/s41467-021-23780-y (PMC8185096; doi:10.1038/s41467-021-23780-y)
Supplement: Supplementary file 2 — Description of Additional Supplementary Files [file 41467_2021_23780_MOESM2_ESM.docx]

File Name: Supplementary Information
Description: Supplementary Figures 1 to 10 and Supplementary Tables 1 to 7

File Name: Supplementary Data 1

Description: Full list of differentially expressed genes for Supplementary Table 3

File Name: Supplementary Data 2

Description: Full list of differentially accessible genes for Supplementary Table 5

File Name: Source Data

Description: Quantitative data of the figures
