# Supplementary material for: Temporal evolution of cellular heterogeneity during the progression to advanced AR-negative prostate cancer
Source: Nat Commun. 2021 Jun 7;12:3372. doi: 10.1038/s41467-021-23780-y (PMC8185096; doi:10.1038/s41467-021-23780-y)
Supplement: Supplementary file 5 — Reporting Summary [file 41467_2021_23780_MOESM5_ESM.pdf]

nature research

Corresponding author(s): David S. Rickman

Last updated by author(s): Apr 6, 2021

Reporting Summary

Nature Research wishes to improve the reproducibility of the work that we publish. This form provides structure for consistency and transparency in reporting. For further information on Nature Research policies, see our Editorial Policies and the Editorial Policy Checklist.

Statistics

For all statistical analyses, confirm that the following items are present in the figure legend, table legend, main text, or Methods section.

n/a	Confirmed	
☐	☒	The exact sample size (*n*) for each experimental group/condition, given as a discrete number and unit of measurement
☐	☒	A statement on whether measurements were taken from distinct samples or whether the same sample was measured repeatedly
☐	☒	The statistical test(s) used AND whether they are one- or two-sided<br>*Only common tests should be described solely by name; describe more complex techniques in the Methods section.*
☒	☐	A description of all covariates tested
☐	☒	A description of any assumptions or corrections, such as tests of normality and adjustment for multiple comparisons
☐	☒	A full description of the statistical parameters including central tendency (e.g. means) or other basic estimates (e.g. regression coefficient) AND variation (e.g. standard deviation) or associated estimates of uncertainty (e.g. confidence intervals)
☐	☒	For null hypothesis testing, the test statistic (e.g. *F*, *t*, *r*) with confidence intervals, effect sizes, degrees of freedom and *P* value noted<br>*Give P values as exact values whenever suitable.*
☒	☐	For Bayesian analysis, information on the choice of priors and Markov chain Monte Carlo settings
☒	☐	For hierarchical and complex designs, identification of the appropriate level for tests and full reporting of outcomes
☐	☒	Estimates of effect sizes (e.g. Cohen's *d*, Pearson's *r*), indicating how they were calculated

Our web collection on statistics for biologists contains articles on many of the points above.

Software and code

Policy information about availability of computer code

Data collection	HiSeq 4000 Sequencer (Illumina), NovaSeq 6000 Sequencer (Illumina), BX63 Fluorescence/Brightfield Microscope with DP80 Camera (Olympus)
Data analysis	The following open source code/software packages were used to collect and analyze data:<br>FastQC v0.11.8, Trimmomatic v0.36, Bowtie2 v2.3.1, STAR v2.5.3a, Cufflinks v2.2.1, HTSeq v0.9.1, DESeq2 v1.26.0, SAMtools v1.8, MACS2 v2.1.1.20160309, HOMER v4.11.1, BEDtools v2.25.0, deepTools v2.5.2, GSEA v3.0, RStudio v1.2.5033, R v3.6.2, CellRanger v3.1.0, CellRanger-ATAC v1.2.0, Loupe Browser v5.0.0, Monocle3 v0.2.0, Bismark v0.19.0, MethylKit v1.16.1, GATK v4.1.1.0, Annovar v2018Apr16, AnalyzePro v1.0, Prism v9.1.0, velocyto v0.17.17, bcl2fastq v2.20, Haplotype Caller (part of GATK v4.1.1.0).

For manuscripts utilizing custom algorithms or software that are central to the research but not yet described in published literature, software must be made available to editors and reviewers. We strongly encourage code deposition in a community repository (e.g. GitHub). See the Nature Research guidelines for submitting code & software for further information.

Data

Policy information about availability of data

All manuscripts must include a data availability statement. This statement should provide the following information, where applicable:

- Accession codes, unique identifiers, or web links for publicly available datasets
- A list of figures that have associated raw data
- A description of any restrictions on data availability

The sequencing data generated in this manuscript have been deposited in the Gene Expression Omnibus (GEO) under accession number GSE151426 [https://www.ncbi.nlm.nih.gov/geo/query/acc.cgi?acc=GSE151426]. Gene sets from the Molecular Signatures Database (MSigDB v7.4) are publicly available [http://www.gsea-msigdb.org/gsea/msigdb/index.jsp] Source data underlying Fig. 1A-C, 1F, 2B, 3E-H, 4E, 4H, 5C, 5G, 6B, 7A, 7D, 7I, S1C, S1D, S2A, S2B, S2F, S2G, S2I, S2J,

S3D, S4H, S6A, S6B, S6D, S7A-C, S9D are available as a Source Data file.

Field-specific reporting

Please select the one below that is the best fit for your research. If you are not sure, read the appropriate sections before making your selection.

[x] Life sciences [ ] Behavioural & social sciences [ ] Ecological, evolutionary & environmental sciences

For a reference copy of the document with all sections, see nature.com/documents/nr-reporting-summary-flat.pdf

Life sciences study design

All studies must disclose on these points even when the disclosure is negative.

Sample size	All sample sizes are included in the relevant figure legends. For animal studies, the sample size was estimated based on our previous experience (Dardenne et al., Cancer Cell, 2016; Berger, Brady, et al., JCI, 2019)
Data exclusions	No data were excluded from analysis.
Replication	All attempts at replication were successful. Specifically, multiple biological replicates were used for RNA-seq, ChIP-seq, single cell RNA-seq, single cell ATAC-seq, and methylation studies. Specific numbers of replications are included in the relevant figure legends.
Randomization	No method of randomization was used. Male littermates were used for mouse experiments. Biological replicates of sequencing-based assays were performed in separate experiments at different times to control for co-variates. Randomization is not applicable to cell culture experiments.
Blinding	All histological evaluations and quantifications (including hematoxylin and eosin (H&E)-stained and IHC images) were performed by a board-certified, genitourinary pathologist (Dr. Brian Robinson) who was blinded to animal genotypes and follow criteria that have previously been described. For other experiments, investigators were not blinded during data collection or analysis.

Reporting for specific materials, systems and methods

We require information from authors about some types of materials, experimental systems and methods used in many studies. Here, indicate whether each material, system or method listed is relevant to your study. If you are not sure if a list item applies to your research, read the appropriate section before selecting a response.

Materials & experimental systems

n/a	Involved in the study	
[ ]	[x]	Antibodies
[ ]	[x]	Eukaryotic cell lines
[x]	[ ]	Palaeontology and archaeology
[ ]	[x]	Animals and other organisms
[ ]	[x]	Human research participants
[x]	[ ]	Clinical data
[x]	[ ]	Dual use research of concern

Methods

n/a	Involved in the study	
[ ]	[x]	ChIP-seq
[x]	[ ]	Flow cytometry
[x]	[ ]	MRI-based neuroimaging

Antibodies

Antibodies used	anti-N-Myc [WB/ChIP-seq] (Santa Cruz: sc-53993), anti-N-Myc [IHC] (Cell Signaling: 51705), anti-EZH2 (BD Bioscience: 612667), anti-GAPDH (Millipore: AB2302), anti-RB1 (Abcam: ab181616), anti-AR (Abcam: ab108341), anti-INSM (Santa Cruz: sc-377428), anti-Keratin 8 (Developmental Studies Hybridoma Bank: TROMA-I), anti-CHGA (Immunostar: 20085), anti-NKX2.1 (ThermoFisher: MS-699), anti-PTEN (Cell Signaling: 9188), anti-POU2F3 (Santa Cruz: sc-293402), anti-mouse IgG (Santa Cruz: sc-2025), anti-mouse IgG-HRP (Cell Signaling: 7076), anti-rabbit IgG-HRP (Cell Signaling: 7074), anti-chicken IgY-HRP (Abcam: ab97135).
Validation	All antibodies are validated by the manufacturer and information can be found at the following websites: [https://www.scbt.com/p/n-myc-antibody-b8-4-b; https://www.cellsignal.com/products/primary-antibodies/n-myc-d4b2y-rabbit-mab/51705; https://www.citeab.com/antibodies/2411662-612667-bd-transduction-laboratories-purified-mouse; https://www.emdmillipore.com/US/en/product/Anti-GAPDH-Antibody,MM_NF-AB2302; https://www.abcam.com/rb-antibody-epr17512-ab181616.html; https://www.abcam.com/androgen-receptor-antibody-er1792-chip-grade-ab108341.html; https://www.scbt.com/p/insm1-antibody-c-1; https://dshb.biology.uiowa.edu/TROMA-I; http://www.immunostar.com/shop/antibody-catalog/sp-1-chromogranin-a-bovine-antibody/; http://tools.thermofisher.com/content/sfs/brochures/D11952~.pdf; https://www.cellsignal.com/products/primary-antibodies/pten-d4-3-xp-rabbit-mab/9188; https://www.scbt.com/p/pou2f3-antibody-6d1; https://www.scbt.com/p/normal-mouse-igg; https://www.cellsignal.com/products/secondary-antibodies/anti-mouse-igg-hrp-linked-antibody/7076; https://www.cellsignal.com/products/secondary-antibodies/anti-rabbit-igg-hrp-linked-antibody/7074; https://www.abcam.com/goat-chicken-igy-hl-hrp-ab97135.html]

Eukaryotic cell lines

Policy information about cell lines

Cell line source(s)	LNCaP cells were obtained from ATCC.
Authentication	Parental LNCaP cells versus N-Myc-expressing cells were assessed by Western blot. Response to androgen stimulation was confirmed by qRT-PCR for AR target gene expression and by RNA-seq. Cell line authenticity was confirmed by STR DNA analysis
Mycoplasma contamination	All cells were checked monthly for mycoplasma contamination and found to be negative.
Commonly misidentified lines (See ICLAC register)	None of the cell lines used are in the ICLAC register.

Animals and other organisms

Policy information about studies involving animals; ARRIVE guidelines recommended for reporting animal research

Laboratory animals	All lines of mice were bred on the same mixed genetic background (C57BL6/129x1/SvJ) and male mice of different ages were analyzed as appropriate (6 and 8 weeks for single-cell studies; between 8 and 52 weeks for tumor studies). Male 6-week-old nude mice (NU/J) were purchased from Jackson Laboratories (Stock #: 002019).
Wild animals	The study did not involve wild animals.
Field-collected samples	The study did not involve samples collected from the field.
Ethics oversight	Mice were maintained and all procedures were performed on male mice following protocols approved by the WCM-IACUC (protocol no. 2008-0019).

Note that full information on the approval of the study protocol must also be provided in the manuscript.

Human research participants

Policy information about studies involving human research participants

Population characteristics	Tumor specimens were obtained prospectively through Institutional Review Board (IRB)-approved protocol with informed consent (WCM IRB no. 1305013903 and 1210013164) or retrospectively (WCM IRB no. 0905010441). For these studies, 29 benign prostate tissues, 66 localized prostate cancers, 73 metastatic castration-resistant prostate adenocarcinomas, and 36 metastatic neuroendocrine prostate cancers were selected for further analysis. All patients were male and tumors were classified based on histomorphology using a published pathologic classification system [Epstein et al., Am. J. Surg. Pathol. 38, 756-767 (2014)]. No other covariates were assessed.
Recruitment	Patients with a prostate cancer diagnosis were cared for at New York Presbyterian Hospital / Weill Cornell Medicine and consented to research use of tumor specimens.
Ethics oversight	Weill Cornell Medicine IRB - Protocol #: 0905010441, 1305013903 and 1210013164.

Note that full information on the approval of the study protocol must also be provided in the manuscript.

ChIP-seq

Data deposition

☒ Confirm that both raw and final processed data have been deposited in a public database such as GEO.

☒ Confirm that you have deposited or provided access to graph files (e.g. BED files) for the called peaks.

Data access links *May remain private before publication.*	https://www.ncbi.nlm.nih.gov/geo/query/acc.cgi?acc=GSE151424
Files in database submission	Raw sequencing reads (.fastq) and Bedgraph (.bdg) of ChIP-seq data.
Genome browser session (e.g. UCSC)	https://www.ncbi.nlm.nih.gov/geo/query/acc.cgi?acc=GSE151424

Methodology

Replicates	N-Myc ChIP-seq was performed in biological replicates using samples from independent mouse tumors.
Sequencing depth	For N-Myc ChIP-seq, 30-35 million single-end reads were obtained for each replicate, resulting in 20-25 million uniquely-mapped reads.

Antibodies	mouse anti-N-Myc (Santa Cruz #: sc-53993, RRID: AB_831602)
Peak calling parameters	ChIP-seq peaks were called using MACS2 with default parameters and a q-value threshold of 0.0001. Sequencing reads from sonicated input chromatin derived from each individual cell line was used as a control for peak calling in all conditions.
Data quality	Quality control of raw sequencing reads was performed using FastQC (Babraham Bioinformatics). Low-quality reads were removed using Trimmomatic with a sliding window size of 4bp and a quality threshold of 20. The resulting reads were aligned to GRCm38 using Bowtie2. PCR duplicates introduced during library creation were removed using SAMtools. Specificity of N-Myc ChIP-seq was confirmed using parental LNCaP cells that do not express N-Myc, which revealed extremely low levels of background enrichment.
Software	FastQC v0.11.8 (https://www.bioinformatics.babraham.ac.uk/projects/fastqc/; RRID:SCR_014583)<br>Trimmomatic v0.36 (http://www.usadellab.org/cms/?page=trimmomatic; RRID:SCR_011848)<br>Bowtie2 v2.3.1 (http://bowtie-bio.sourceforge.net/bowtie2/; RRID:SCR_016368)<br>SAMtools v1.8 (http://www.htslib.org/doc/samtools-1.4.html; RRID:SCR_002105)<br>MACS2 v2.1.1.20160309 (https://github.com/taoliu/MACS)<br>BEDtools v2.25.0 (http://bedtools.readthedocs.io/en/latest/index.html; RRID:SCR_006646)<br>HOMER v4.11.1 (http://homer.ucsd.ede/homer/introduction/programs.html)<br>deepTools v2.5.2 (https://deeptools.readthedocs.io/en/latest/index.html; RRID:SCR_016366)<br>GSEA v3.0 (http://software.broadinstitute.org/gsea/index.jsp; RRID:SCR_003199)
